# Loci under selection and markers associated with host plant and host-related strains shape the genetic structure of Brazilian populations of *Spodoptera frugiperda* (Lepidoptera, Noctuidae)

**DOI:** 10.1371/journal.pone.0197378

**Published:** 2018-05-22

**Authors:** Karina Lucas Silva-Brandão, Aline Peruchi, Noemy Seraphim, Natália Faraj Murad, Renato Assis Carvalho, Juliano Ricardo Farias, Celso Omoto, Fernando Luis Cônsoli, Antonio Figueira, Marcelo Mendes Brandão

**Affiliations:** 1 Centro de Energia Nuclear na Agricultura, Universidade de São Paulo, Campus "Luiz de Queiroz", Laboratório de Melhoramento de Plantas, Piracicaba, São Paulo, Brazil; 2 Instituto Federal de Educação, Ciência e Tecnologia de São Paulo, campus Campinas CTI Renato Archer, Campinas, São Paulo, Brazil; 3 Programa de Pós-graduação em Genética e Biologia Molecular, Centro de Biologia Molecular e Engenharia Genética, Universidade Estadual de Campinas, Campinas, São Paulo, Brazil; 4 Monsanto do Brasil Ltda, São Paulo, São Paulo, Brazil; 5 Instituto Phytus, Departamento de Entomologia, Rua Duque de Caxias, Santa Maria, Rio Grande do Sul, Brazil; 6 Escola Superior de Agricultura "Luiz de Queiroz", Universidade de São Paulo, Departamento de Entomologia e Acarologia, Piracicaba, São Paulo, Brazil; 7 Centro de Biologia Molecular e Engenharia Genética, Universidade Estadual de Campinas, Campinas, São Paulo, Brazil; University of Tennessee, UNITED STATES

## Abstract

We applied the ddRAD genotyping-by-sequencing technique to investigate the genetic distinctiveness of Brazilian populations of the noctuid moth *Spodoptera frugiperda*, the fall armyworm (FAW), and the role of host-plant association as a source of genetic diversification. By strain-genotyping all field-collected individuals we found that populations collected from corn were composed primarily of corn-strain individuals, while the population collected from rice was composed almost entirely of rice-strain individuals. Outlier analyses indicated 1,184 loci putatively under selection (ca. 15% of the total) related to 194 different Gene Ontologies (GOs); the most numerous GOs were nucleotide binding, ATP binding, metal-ion binding and nucleic-acid binding. The association analyses indicated 326 loci associated with the host plant, and 216 loci associated with the individual strain, including functions related to *Bacillus thuringiensis* and insecticide resistance. The genetic-structure analyses indicated a moderate level of differentiation among all populations, and lower genetic structure among populations collected exclusively from corn, which suggests that the population collected from rice has a strong influence on the overall genetic structure. Populations of *S*. *frugiperda* are structured partially due to the host plant, and pairs of populations using the same host plant are more genetically similar than pairs using different hosts. Loci putatively under selection are the main factors responsible for the genetic structure of these populations, which indicates that adaptive selection on important traits, including the response to control tactics, is acting in the genetic differentiation of FAW populations in Brazil.

## Introduction

Studies of ecological speciation [1, 2] comprise the investigation of the mechanisms of reproductive isolation among populations caused by divergent selection [2, 3]. An important prediction of ecological speciation is that pairs of populations of herbivores using different host plants will be more reproductively isolated than pairs using the same host plant, because ecological divergence is an indication of divergent selection [4, 5]. This pattern of positive correlation between adaptive phenotypes and population divergence, independent of the genetic distance, is called "isolation by adaptation" [6, 7]. Ecological speciation associated with the use of larval host plants has been extensively studied, mostly due to the intimate relationship between herbivorous insects and their host plants, as both a food resource and oviposition site [8]. While the classic example of host speciation is the fly *Rhagoletis pomonella* Walsh (Diptera, Tephritidae) [9–11], ecological differentiation followed by genetic divergence associated with food resources has been demonstrated for several species of lepidopteran insects [12–15].

If divergence among populations is due to genetic drift and host-independent selection, gene flow among populations would be estimated by genetic distance in neutral loci [4]. On the other hand, if divergence is adaptive to the use of host plants, populations using the same food plant will be genetically divergent in key sites of their genomes. In this case, it is necessary to quantify the distinction among populations in relation to their local adaptation, which also can involve a few genes with key functions [16]. In accordance with this model of "divergence-with-gene-flow" [17, 18], populations would diverge at some genetic regions due to natural selection, while other loci would share variations due to historic or recent gene flow, among other reasons [7, 19]. Adaptive responses to different host plants can impose selective divergent pressures on digestive and physiological characteristics related to the process of metabolization of chemical compounds, for instance [1]. The challenge is to identify ecologically important genes under selection, that are involved in the process of differentiation and speciation [20, 21].

The emerging area of population genomics aims to identify these genes, and the use of Next Generation Sequencing (NGS) techniques has made possible studies of population genomics with data comprising all the genome information of organisms [16, 21–23]. Here we used the ddRAD genotyping-by-sequencing technique [24–26] to characterize the genetic distinctiveness of populations of the polyphagous noctuid moth *Spodoptera frugiperda* (J. E. Smith), the fall armyworm (FAW), throughout its distribution in Brazil, and to investigate the role of host-plant association as source of genetic structure of field populations of FAW. Genotyping-by-sequencing has been widely applied in population genetics studies of insects in recent years [27–34]. The technique presents several advantages over more often used markers; the main advantage is the possibility to investigate genetic regions under selection related to ecological features, such as the preference for host plants.

*Spodoptera frugiperda* is the most important pest of corn (*Zea mays* L.) in South America [35], and is found throughout Brazil as a pest of corn and several other crops, such as rice (*Oryza sativa* L.), and cotton (*Gossypium hirsutum* L.). Pest control of FAW is made by using both *Bacillus thuringiensis* genetically modified corn (*Bt*-corn) and insecticides [36].

The FAW is differentiated into host plant-related strains with their own ecological, genetic and physiological features [37]. One strain feeds preferentially on corn, sorghum and cotton (corn strain, CS), while the other usually feeds on rice and other pasture grasses (rice strain, RS) [38], although host plant fidelity is not absolute. This dissimilarity is not complete, and evidence of hybridization between the strains has been presented [39]. Although hardly distinguished morphologically, except by differences in wing morphometrics [40], the two strains show evidence of reproductive isolation, such as differences in the female pheromone composition [41], and in the period of reproductive activity [42], fertility loss due to interbreeding between strains [43], and assortative mating [44]. There are evidences that CS and RS individuals may also differ in their tolerances to *Bt* toxins [45] and to certain insecticides [46, 47]. Recent studies have gone so far as to suggest that corn and rice strains of FAW should have the status of sibling species, based on post-zygotic reproductive isolation between the two strains [48]. All these differences are suggested to be a consequence of the preferential use of host plants in field conditions [39]. In southern Brazil, AFLP markers implied differentiation among populations according to the host plant used [49], and a larger geographic sampling in South America revealed population structures related to host plant rather than to geographical origin, although other factors must be acting to maintain genetic differentiation [50]. Genetic differentiation in FAW populations as result of their association with host plants was also suggested in Tolima, Colombia [51]. Significant variation between the corn and rice strains in the number of digestion and detoxification genes, found in a comparative genome study, also indicates differential adaptation to alternative host plants [52].

Taken together, these studies point to a speciation process in populations of *S*. *frugiperda* [53, 54]. By applying ddRAD markers to study Brazilian populations of FAW, we aimed to answer several questions: (1) are these populations structured according to the host plant where they were collected? (2) are pairs of populations using the same host plant genetically more similar than pairs of populations using different host plants? (3) which loci are putatively under selection in these populations? (4) are either "neutral" loci or loci "under selection" responsible for genetic structure in these populations? Additionally, loci under selection and those associated with host plants and strains were thoroughly investigated to improve our knowledge of the mechanisms responsible for the interactions among the populations of FAW.

## Material and methods

### Sampling

**Fig 1:**
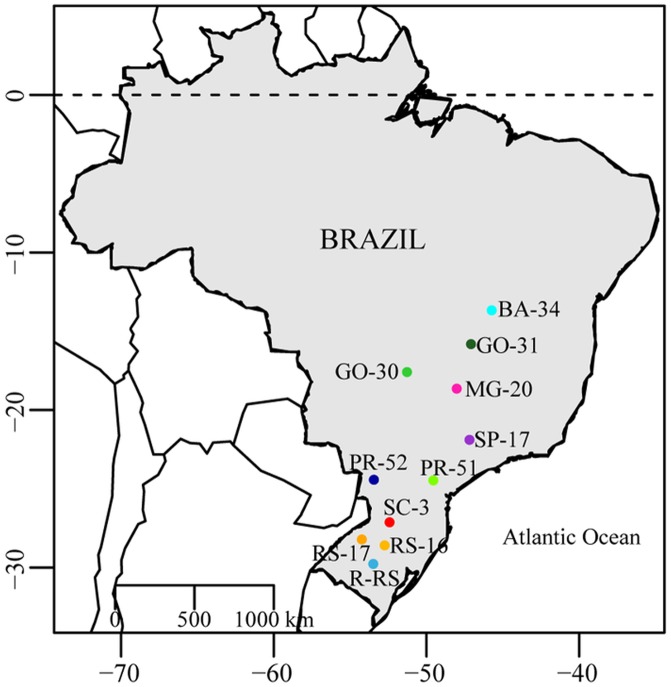
Map showing all sampled populations of *S*. *frugiperda*. Origin of populations of corn- and rice-strain larvae of *S*. *frugiperda*. The map was constructed using the R package, with the libraries map, mapdata, and maptools.

A total of 329 individuals of *S*. *frugiperda* were collected on non-*Bt*-corn (refuge areas) and rice in 11 localities throughout Brazil, separated by distances between 151 and 1,957 Km (Fig 1, Table 1). Genetic property was registered under SISGEN #ACF86DD. Larvae were collected by hand early in the infestations, and were reared to the pupa stage on a white bean-based artificial diet (adapted from [55]). Pupae were placed in Petri dishes lined with filter paper and covered with a thin layer of vermiculite until adult emergence. Recently emerged adults were immediately frozen at –20 °C.

**Table 1:** Collection data of samples of *S*. *frugiperda*.

Code	Locality (city, state)	Date	Latitude (S)	Longitude (W)	Host Plant	N
**R-RS**	Santuário, Restinga Seca, RS	Jan 14, 2015	29.77236	53.48369	Rice	33
**BA-34**	Correntina, BA	Dec 27, 2013	13.6755	45.7270	Corn	30
**GO-30**	Jataí, GO	Dec 27, 2013	17.5941	51.2730	Corn	30
**GO-31**	Cabeceiras, GO	Jan 21, 2014	15.82108	47.08283	Corn	30
**MG-20**	Araguari, MG	Nov 18, 2013	18.645472	48.018111	Corn	30
**PR-51**	Castro, PR	Dec 10, 2013	24.4715	49.5490	Corn	29
**PR-52**	Cascavel, PR	Dec 14, 2013	24.4310	53.4435	Corn	30
**RS-16**	Não me Toque, RS	Dec 6, 2013	28.59515	52.73720	Corn	30
**RS-17**	Santo Ângelo, RS	Dec 6, 2013	28.21909	54.23113	Corn	30
**SC-3**	Seara, SC	Dec 14, 2013	27.12998	52.41587	Corn	30
**SP-17**	Casa Branca, SP	Nov 13, 2013	21.8951	47.1825	Corn	30

Sampled populations of *S*. *frugiperda*. N = number of individuals collected from each site.

### DNA extraction

Total genomic DNA was extracted from thoracic tissue using the CTAB protocol [56]. Thoracic tissue was homogenized in 650 μL CTAB buffer with 0.2% β-mercaptoethanol and 0.05% proteinase-K, and incubated for 1 h at 55 °C. One volume of chloroform:isoamyl alcohol (24:1) was added to the homogenate and the mixture was centrifuged at 13,000 rpm for 5 min at 4 °C. The supernatant was removed, transferred to a new tube, and extracted with one volume of chloroform:isoamyl alcohol (24:1) and 200 μL CTAB buffer; the mixture was centrifuged as before, the supernatant was removed and transferred to a new tube, and the extraction was repeated once more. DNA was precipitated by adding 650 μL ice-cold isopropyl alcohol to the aqueous phase, followed by incubation for at least 1 h at −20 °C. After incubation, the precipitate was centrifuged at 13,000 rpm for 5 min at 4 °C. The isopropyl alcohol was discarded and the pellet was rinsed once with ice-cold 70% ethanol, followed by centrifugation for 20 min. The ethanol was discarded and the pellet was allowed to dry at room temperature. The DNA was eluted in 40–50 μL EB buffer (10 mM Tris pH 8.0) and stored at −20°C. Each sample was run in a 1% agarose gel in SB 1X buffer (10 mM sodium hydroxide, pH adjusted to 8.5 with H_3_BO_3_) to confirm sample quality. The DNA concentration and 280/260 and 260/230 nm ratios were estimated by UV absorption in a NanoDrop spectrophotometer (Techno Scientific, Wilmington, DE, USA). Next, the final concentration was estimated in a Qubit^®^ 2.0 (Thermo Scientific, Waltham, MA, USA), and the amount of DNA per sample was normalized to 20 ng/μL.

### Strain identification

The strain of each field-collected individual was identified using the strain-specific *Msp*I site in the mitochondrial gene cytochrome c oxidase subunit I (COI). The *ca*. 569-bp fragment of COI was amplified using the primers JM76 and JM77 [57]. Reactions were carried out in 25 μL total volume, using 1 μL genomic DNA, 2 mM MgCl_2_, 40 μM dNTPs, 0.2 μM of both forward and reverse primers, 1U GoTaq DNA Polymerase (Promega, Fitchburg, WI, USA), 10% volume of 10X *Taq* buffer and 10% volume of 5% dimethyl sulfoxide (DMSO). The PCR program included an initial incubation at 94 °C (2 min), followed by 38 cycles of 94 °C (45 s), 56 °C (45 s), 72 °C (1 min), and a final incubation of 72 °C for 5 min. After amplification, 1.0 μL of FastDigest *MspI* (Thermo Scientific) was added to 10 μL of each reaction, incubated at 37 °C for 10 min, and the complete volume was loaded in a 2% agarose gel in TAE buffer (40 mM Tris, 20 mM acetic acid, 1 mM EDTA, pH 8.0).

### Libraries construction and sequencing

Genotyping-by-sequencing libraries were constructed using standard protocols [26], with minor modifications, at the *Plateforme d’Analyses Génomiques of the Institut de Biologie Intégrative et des Systèmes* (IBIS, Université Laval, Québec city, Canada). Total genomic DNA (200 ng) was simultaneously digested with both high-fidelity *Pst*I (New England Biolabs, Ipswich, MA, USA) and *Msp*I (New England Biolabs). One of 96 barcoded adapters was ligated on the *Pst*I cut site for each individual sample, and a common adapter (adapter 2) was ligated onto the *Msp*I cut site of all samples with T4 ligase (New England Biolabs). All 96 samples (same volume) were pooled and size-selected using a 2% agarose gel cassette on a BluePippin instrument (SAGE Science, Beverly, MA, USA) with the elution time set from 50 to 65 min. Eluted fragments were used for multiplexed PCRs, using standard forward primer A and reverse primer C [26]. Final libraries were checked for quality on a High Sensitivity BioAnalyzer chip (Agilent, Santa Clara, CA, USA) and quantified using Picogreen (Thermo Fisher). Each library with 96 samples each was sequenced in two lanes of an Illumina HiSeq2000 (Illumina, Inc., San Diego, CA, USA) using 100-bp single-end reads, at the McGill University and Génome Québec Innovation Centre (Montreal, Canada).

### Demultiplexing and SNP calling

Samples demultiplexing and SNP calling were performed using a pipeline with specific parameters on the software Stacks [58]. A custom workflow was designed to execute all the steps involved in these tasks, enabling node parallel execution where it was possible, on a computer cluster with a queuing system managed by Torque/Maui (Adaptive Computing Enterprises Inc., Provo, UT, USA). Job submission scripts can be downloaded at https://github.com/bioinfo-guy/Stacks_GBS_pipeline.

First, raw sequence reads were demultiplexed and cleaned (process_radtags). Next, the data from each sample were grouped into loci, and polymorphic nucleotide sites were identified, using the package ustacks with no-reference genome (m = 6, M = 2, N = 3, max_locus_stacks = 4, k_len = 14). Cstacks was used to group loci across samples and to create a catalogue (n = 1, k_len = 14). Sstacks was then applied to match each sample against the catalogue in order to define the allelic state at each locus (m = 6, M = 2, N = 3, max_locus_stacks = 4, k_len = 14). Finally, the allelic states were subjected to population-genetics statistics using the package *population* (r = 0.80, p = 3, m = 4, f = p-value, min_maf = 0.05,—write_random_snp) and all possible output files were generated for downstream analyses. When necessary, the data-conversion tool PGDSpider [59] was used to convert input files for specific software.

### Outlier analyses

Lositan [60] was used to detect loci under selection based on the neutral distribution of F_ST_ values for all loci in relation to *He* (expected heterozygosity). We ran the program three times: the first run included all loci under an attempted neutral mean F_ST_, with 50000 simulations, 99% confidence interval, infinite alleles mutation model, and false discovery rate of 0.1%. After the first run, all loci outside the confidence interval were removed and the mean neutral F_ST_ was recalculated to reduce the bias in the estimation of the mean neutral F_ST_ by eliminating extreme loci from the computation [60]. Only the supposed neutral loci were kept in the second run, using the same parameters as above. The third run comprised all loci and the newly calculated neutral F_ST_, with all other parameters maintained. This procedure was repeated three times, and loci recovered as outliers in all three replications were inferred to be under selection.

### Association analyses

To investigate the mechanisms involved in the relationships among the populations of the FAW, we tested the association between loci and two individual features, *host plant* where the individual was collected, and its *strain*. We applied a standard case/control association analysis available in the package Plink v. 1.9 [61], using Bonferroni for adjustment for multiple testing. Input files .ped and .map were created directly from the Stacks package *population*. As we consider that the two features are not independent, since the preference for a host plant is mostly due to the individual strain, we constructed a Venn diagram (http://bioinformatics.psb.ugent.be/webtools/Venn/) using a list of loci independently associated with the two features, to permit us to visualize both the shared loci and the exclusive loci associated with each feature. We used the same approach to sort loci that are simultaneously associated with the two features and are putatively under selection.

### Transcriptome mapping and annotation

All loci putatively under selection and feature-associated loci were mapped on the transcriptome proposed for the FAW (Bioproject: PRJNA408280, Biosample: SAMN07678153) [62], using bowtie2 [63]. All loci mapped to contigs were identified using samtools [64, 65] and stored in a correlational database used to integrate the loci mapping profile and contig annotation.

Previously assembled contigs [62] had their functional annotation updated by a customized set of perl scripts and local databases constructed with publicly available data (all databases are available upon request to the authors). In brief, all contigs were searched by similarity against NCBI REFSEQ [66] (updated on April 23, 2016) and MEROPS v. 9.12 [67] (a specific database for peptidases), using an e-value cutoff of 10 e^−5^ and HSP similarity threshold of 80%. Patterns of RNA families were indicated by hmmscan [68] using RFAM database v. 12 [69]. Patterns of protein families from the PFAM database [70] were proposed by hmmscan, using a set of translated peptides from candidate coding regions within the assembled transcriptome sequences indicated by transdecoder (https://transdecoder.github.io/).

The sequence description was achieved by integrating all database searches. Blast best hit results, from all databases previously described, were designated by a restrictive e-value and HSP similarity cutoff (1 e^–10^ and 90%, respectively) sorted by the latter; RNA and protein families from hmmscan were filtered by the Expectation Value (1 e^–10^) on the full sequence column from the resulting analyses. The gene ontology-controlled vocabulary terms were assigned to all sequences by a custom perl script using all search results, following the thresholds described above and removing all obsolete terms.

Because *S*. *frugiperda* is controlled in the field by both *Bacillus thuringiensis* genetically modified corn (*Bt*-corn) and insecticides [36], we manually enriched the loci related to *Bt* and insecticide resistance [71–74].

### Relationships among individuals based on neutral loci and loci putatively under-selection

We expected that loci under-selection could be more differentiated among populations of FAW than neutral loci, if the ecological speciation model was at least partially responsible for their discrimination in the field. To test the ability of neutral loci and loci under-selection to resolve the relationships among all collected individuals of FAW, we used a Bayesian approach available in Beast v. 2.4.5 [75]. We first converted the .vcf file in .fasta using the script VCF2FASTA (https://github.com/vcflib/vcflib). The .xml files with neutral loci and loci putatively under-selection were created on the BEAUti interface (comprised in the package Beast v. 2.4.5) using the GTR model of nucleotide substitution, clock rate = 1.0 and Yule process of speciation. One MCMC analysis included 50 mi generations (with a pre-burn-in of 20%), storing parameters every 1000 steps. Tracer v. 1.5 (Drummond & Rambaut 2007) was used to examine the ESS of the different parameters and to define the ‘burn in’. TreeAnnotator v. 2.4.5 was used to conduct a 20% ‘burn in’ and to generate a maximum clade credibility topology of all the sampled trees rescaled to match posterior median estimates. Finally, the software FigTree v. 1.3.1 was used to visualize the topology of the Bayesian trees.

### Population-genetics analyses

Genetic structure of FAW populations was estimated by non-hierarchical locus by locus Analysis of Molecular Variance (AMOVA), using the software Arlequin v. 3.5 [76], and parameters Φ were estimated for: (1) all populations, without discriminating host-plant origin; (2) populations collected exclusively from corn; (3) populations collected in Rio Grande do Sul (RS) from both corn and rice, since that is the only locality where it was possible to test the hypothesis that pairs of populations using different host plants are more structured than pairs using the same host plant; and (4) samples characterized as CS or RS according to the strain-specific *Msp*I site in COI. Non-hierarchical analyses were first computed using all loci and then estimated using neutral loci or loci under-selection separately. Hierarchical AMOVA was conducted for two grouping configurations using combined neutral loci and loci under-selection: (1) among all populations collected from the two host plants, corn and rice; and (2) among populations from Rio Grande do Sul (RS) collected from the two host plants. Genetic structure was interpreted from the Φ statistics associated with the different hierarchical levels in which variation is distributed [77]. The significance of the Φ_ST_ values was evaluated using 16000 permutations, computed distance matrix using pairwise difference, and gamma *a* value = 0. Pairwise genetic distances were estimated using Slatkin’s method [78] in Arlequin.

The population structure was also estimated using a network-based approach in the R package Netview P v. 1.0 [79]. Input files .ped and .map were created directly from the Stacks package *population*, as before. First, a genetic-distance matrix of all collected individuals, including strain information and all loci, was computed with Plink v. 1.9 [61]. Netview P was then used to construct a network to detect the community structure, and to visualize the final network topology. The number of mutual nearest neighbors (*k*) was set to 10, and a *k* = 40 was also used to test for large-scale genetic structure.

A Discriminant Analysis of Principal Components (DAPC, [80]) was also applied to provide a visual evaluation of the genetic structure of Brazilian FAW populations. The R package adegenet [81] was applied for DAPC estimations, using sampling localities as prior groups, and all collected individuals and all loci. We applied the same approach to test only populations collected from cornfields. The find.clusters function was applied to identify genetic clusters. This method involves running successive K-means with an increasing number of clusters (k), after transforming data using a principal components analysis (PCA), and the optimal clustering solution corresponded to the lowest Bayesian Information Criterion (BIC). Adegenet was also applied to compute contributions of the alleles to the clustering pattern, with threshold = 0.0008. All alleles above the threshold value were identified by the annotation in the transcriptome as performed before, with special attention to alleles fixed in only one of the genetic clusters. Alleles identified at this step were also manually blasted in SpodoBase [82].

## Results

### Strain identification

**Fig 2:**
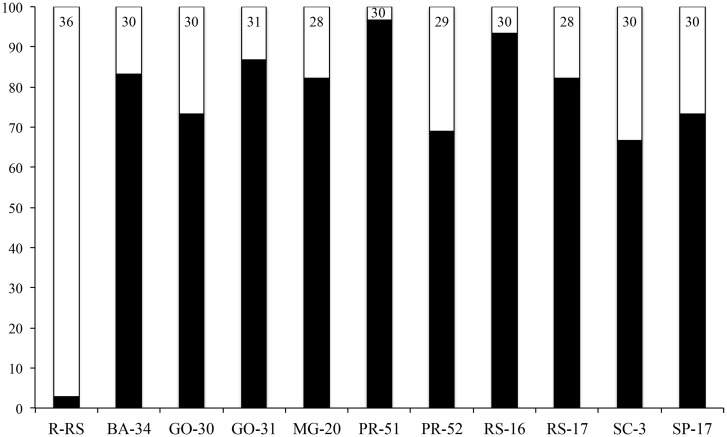
Bar graph showing the frequencies of corn-and rice-strain individuals. Frequency of corn- (CS, black) and rice- (RS, white) strains in field populations of *S*. *frugiperda*. Numbers into the bars indicate the amount of individuals evaluated in each locality.

The corn strain (CS) was more common in all populations collected from corn, according to the strain-specific *Msp*I site in COI, and ranged from 69–97% of the individuals within localities. All but one individual collected from rice (R-RS) were characterized as rice strain (RS) (Fig 2).

### Libraries construction and SNP calling

The number of reads for each run per lane of HiSeq2000 ranged from 142 to 196 million, with 96 samples per library. Each library was run in two lanes. From a total of 1.418.091.728 sequence reads, 87.1% was retained for Stacks analyses, generating a catalog containing 3.073.340 loci. Ustacks presented 3.8464,70 as coverage depth mean among all populations included on this study, with standard deviation of 193.082,3.

The population genetic statistics calculated in Stacks recovered 7,664 SNPs. The population from São Paulo (SP-17) was removed from downstream analyses due to a lack of data, which could increase the bias in posterior analyses, and the final matrix had 269 individuals.

### Outlier analyses

Lositan indicated 1184 putatively under-selection loci (ca. 15% of the total loci). The functional annotation gathered 194 different Gene Ontologies (GOs) using the FAW transcriptome as reference. The most represented GO was nucleotide binding, functionally described as elongation factor Tu GTP binding domain, multidrug resistance-associated protein 1, ABC transporter transmembrane region, and insulin receptor, among other descriptions (S1 Table). Other common GOs included ATP binding, metal-ion binding and nucleic-acid binding (S1 Table). Molecular functions related to metabolization of host plants included UDP-glucuronosyl and UDP-glucosyl transferase, cytochrome P450 (mainly CYP6B7-like) and peptidases. Loci annotated as unigenes related to *Bacillus thuringiensis* (*Bt*) resistance included zinc carboxypeptidases and ABC transporters (loci 12150 and 19063). The two contigs where these ABC loci were mapped (contigs 12636 and 28035, respectively, [62]) showed high similarity with ABC genes involved in *Bt* resistance in other species of Lepidoptera, such as ABCC2 and ABCC3 of *Spodoptera exigua* [83, 84], and ABCA2 of *Helicoverpa armigera* [85], and ABCB1 of the leaf beetle *Chrysomela tremula* [86], including the regions of transporter motifs (TpM1 and TpM2) and ATP-binding (ATP1 and ATP2) (S1 and S2 Figs). Loci annotated as genes known for their role in insecticide resistance [74] included unigenes related to glutathione transferase activity, cytochrome P450 (mainly 6B7-like), and carboxylesterase (S1 Table). Three GOs were described as ryanodine receptors (related to calcium and ion channel activities), which are known as the molecular target-site of diamide insecticides [87]; diamides have been widely applied to control cornfield populations of FAW [88]. Three unigenes putatively under selection were also described as down- or up-regulated in the saliva of corn-strain larvae of *S*. *frugiperda* [89]: ecdysone oxidase, arginine kinase, and translation elongation factor.

### Association analyses

**Fig 3:**
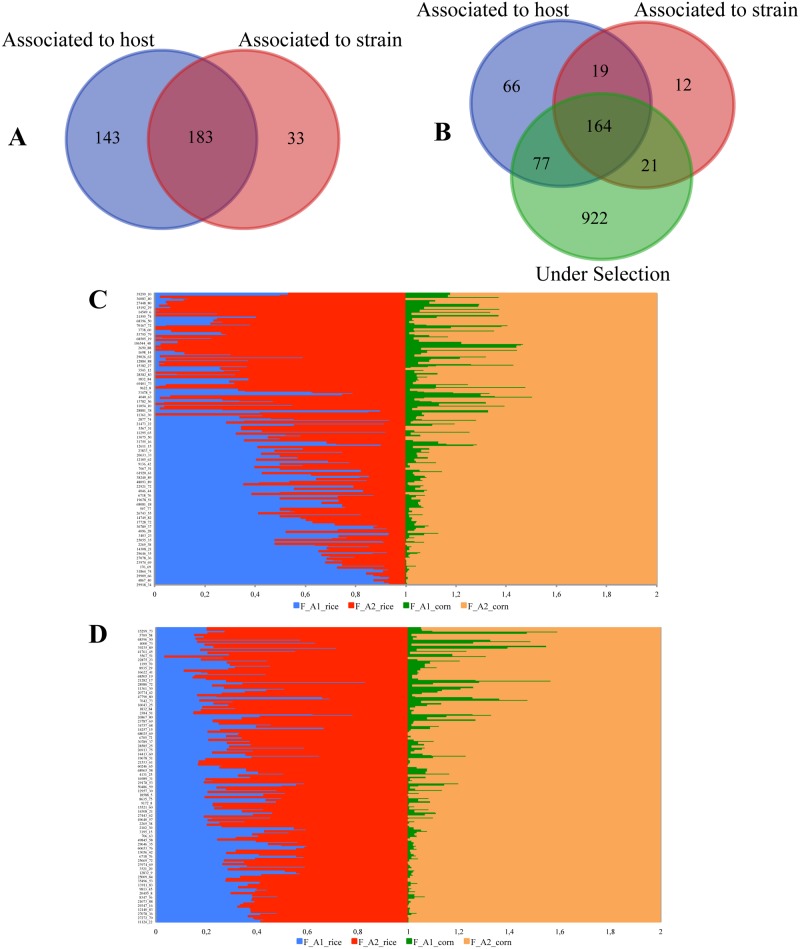
Association analyses. A) Number of loci associated either to the host plant where the individual was collected or to the individual strain; B) Number of loci under selection associated to individual host plant and strain; C) Frequency of alleles of 326 loci significantly associated to host plant; F_A1_rice and F_A2_rice are the frequencies of alleles A1 and A2 for each loci in the population sampled on rice, considering that the FAW is diploid and freqA1+freqA2 = 1; F_A1_corn and F_A2_corn are the frequencies of alleles A1 and A2 for each loci in the populations sampled on corn; D) Frequency of alleles of 216 loci significantly associated to individual strain; F_A1_rice and F_A2_rice are the frequencies of alleles A1 and A2 for each loci in the rice strain individuals; F_A1_corn and F_A2_corn are the frequencies of alleles A1 and A2 for each loci in the corn strain individuals.

The association analyses indicated 326 loci significantly associated with the host plant where the individual was collected, and 216 loci significantly associated with the individual strain. One hundred forty-three (143) loci were exclusively associated with host plant, 33 with strain, and 183 were simultaneously associated with both features (Fig 3A). One hundred sixty-four (164) loci are both putatively under selection and associated with both features (Fig 3B). Around 14–20% of the loci were successfully annotated using the transcriptome of *S*. *frugiperda* as reference (S2 Table). One locus significantly associated with the host plant (locus 15958) was annotated as a zinc carboxypeptidase, and one locus significantly associated with the strain (locus 8935) was annotated as cadherin-related tumor suppressor; both molecular functions are known to be related to *Bt*-resistance in insects. Locus 8935 was mapped to the contig 63619 in the FAW transcriptome; when the contig 63619 was blasted against the NCBI data bank, two sequences were indicated with the highest similarity: a predicted *Spodoptera litura* cadherin-related tumor suppressor (LOC111348058) (identities 959/1012, 94%), and a predicted *Helicoverpa armigera* cadherin-related tumor suppressor (LOC110371604) (identities 853/1011, 84%). The same contig showed the highest identity with the scaffold 9577 (96%) when blasted against the corn variant assembly 3.1 of the genome of *S*. *frugiperda* (https://bipaa.genouest.org/is/lepidodb/spodoptera_frugiperda/), and with the SFRU RICE 028070 when blasted against the rice variant assembly 1.0 (96% identity). The alignment of the contig 63619 with several cadherin sequences available did not permit to determine if it is in any important region related to *Bt* resistance (S3 Fig). Locus 29602 was simultaneously associated with the host plant and strain, and it was annotated as glutathione transferase, which is highly related to insecticide resistance in insects [74, 90]. Locus 5033 was also associated with both features and it was annotated as insulin receptor, which plays an important role in feeding behavior in insects [91]. All loci simultaneously associated with the host plant and strain, that were able to be annotated, are also putatively under selection according to our outliner analyses.

Many loci significantly associated with the host plant have one allele fixed in individuals collected from rice (Fig 3C), while this pattern was not seen in loci associated with strains (Fig 3D). According to these results, loci associated with the rice strain are more polymorphic than loci associated with rice plants. Loci associated with the corn strain or corn plants in the field in general showed many fixed loci and low polymorphism (Fig 3C and 3D).

### Relationships among individuals based on neutral loci and loci putatively under-selection

**Fig 4:**
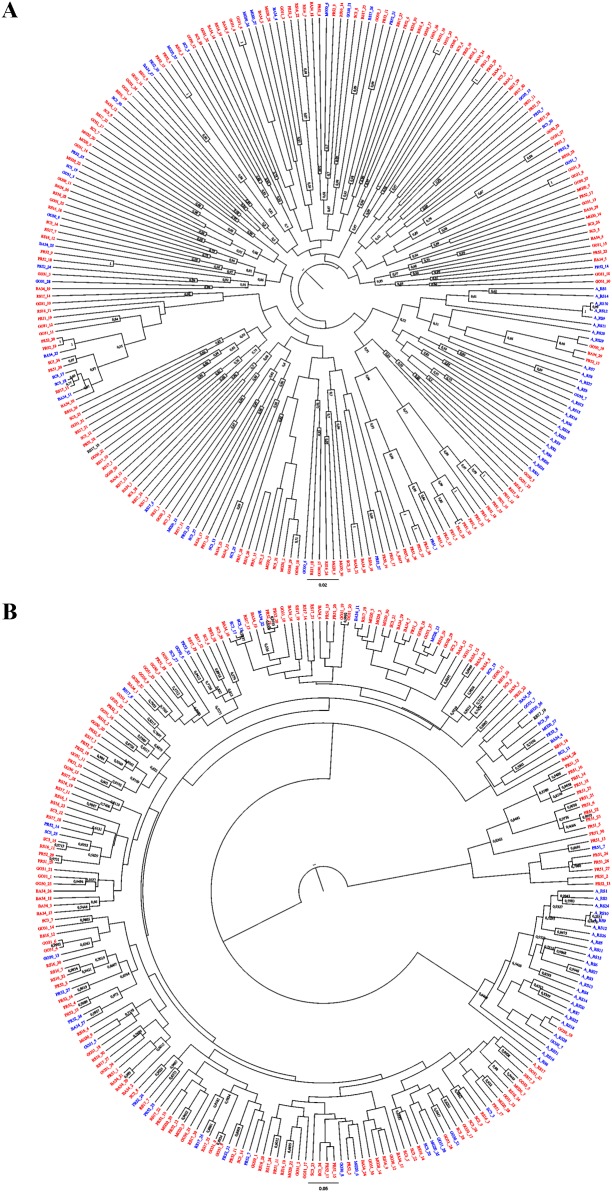
Bayesian trees. Topologies indicating relationships among individuals of *S*. *frugiperda* (FAW) based on A) neutral loci and B) loci under selection. Numbers on the branches indicate posterior probability values above 0.50. Corn-strain individuals are indicated in red letters, and rice-strain individuals in blue.

The tree topology recovered with loci under-selection was better resolved than the topology recovered with neutral loci (Fig 4A and 4B). It is possible to distinguish three main clades in the tree obtained with loci under-selection (Fig 4B): one composed almost exclusively of rice-strain individuals from Rio Grande do Sul (R-RS), another composed mostly of corn-strain individuals from Paraná (PR-51), and a more variable clade composed of corn- and rice-strain individuals from all other populations. Individuals of the same population were not always grouped together in the same clade, whether or not neutral loci or loci under-selection were used as input.

### Population-genetics analyses

The genetic structure of Brazilian populations of FAW estimated by AMOVA of all populations, including all loci, indicated a moderate level of differentiation, with F_ST_ = 0.056. When only neutral loci were considered, F_ST_ value was reduced by half (0.028). Conversely, when we considered only loci under-selection, the value of F_ST_ increased 10× in relation to neutral loci (0.287) (Table 2). For populations collected from corn, the genetic structure was lower considering either all loci or neutral loci and loci under-selection separately, which suggests that the population collected from rice has a strong influence on the genetic structure of the populations of FAW that we collected in Brazil. For populations from Rio Grande do Sul (RS), all loci indicated moderate levels of genetic structure (F_ST_ = 0.096), but the structure decreased when we considered only neutral loci, and was strongly increased when only loci under-selection were considered. Genetic structure was the lowest when we consider individual strains of FAW (Table 2).

**Table 2:** AMOVA.

	Source and percentage of variation		
	Among populations	Within populations	F_ST_
All populations, all loci	5.59	94.41	0.056
All populations, neutral loci	2.75	97.24	0.028
All populations, loci under selection	28.72	71.28	0.287
Populations from corn, all loci	2.66	97.34	0.027
Populations from corn, neutral loci	2.21	97.79	0.022
Populations from corn, loci under selection	7.38	92.62	0.074
Populations from RS, all loci	9.61	90.38	0.096
Populations from RS, neutral loci	3.86	96.14	0.038
Populations from RS, loci under selection	39.96	60.04	0.400
Corn (CS) and rice (RS) strains, all loci	1.16	98.84	0.012

Non-hierarchical AMOVA considering either all loci or neutral and loci under selection separately. All significance tests of F_ST_ values resulted in p < 0.001.

Hierarchical AMOVA indicated that most of the variation is within populations, when either all populations or only populations from Rio Grande do Sul were considered. Values of F_ST_ were similar for the two sets of populations (Table 3).

**Table 3:** Hierarchical AMOVA.

	Source e percentage of variation					
	Amongrice *vs* corn	Among populations within groups	Within populations	F_ST_	F_SC_	F_CT_
All populations	13.65	2.22	84.13	0.159*	0.026*	0.136*
Populations from RS	10.91	1.73	87.36	0.126*	0.019**	0.109*

Hierarchical AMOVA considering all loci.

Significance tests of F_ST_:

* p < 0.001

or

** p < 0.05.

Pairwise Slatkin´s F_ST_ values were higher among the population collected from rice in Rio Grande do Sul (R-RS) and all other populations, including the other two populations collected in Rio Grande do Sul from corn (Table 4). F_ST_ values were also high among population PR-51 from Castro, Paraná, and all other populations, including the closest population from Paraná (PR-52). Pairwise structure was low among all other populations (Table 4).

**Table 4:** Pairwise Slatkin´s F_ST_.

	R-RS	BA-34	GO-30	GO-31	MG-20	PR-51	PR-52	RS-16	RS-17
R-RS	-								
BA-34	**0.82161**	-							
GO-30	**0.75060**	0	-						
GO-31	**0.84697**	0	0.00083	-					
MG-20	**0.69477**	0.00619	0	0.00901	-				
PR-51	**0.90921**	**0.11205**	**0.14882**	**0.11105**	**0.19961**	-			
PR-52	**0.83186**	0	0	0.00979	0.01646	**0.11665**	-		
RS-16	**0.80248**	0.00386	0.00408	**0.02834**	0.02205	**0.14273**	0	-	
RS-17	**0.74009**	0	0	0.01006	0	**0.13886**	0	0	-
SC-3	**0.79219**	0.00977	0.00588	0.01040	0.01394	**0.13671**	0	0.01052	0

Pairwise Slatkin´s F_ST_ for all populations, considering all loci. Bold values are significant (p < 0.05).

**Fig 5:**
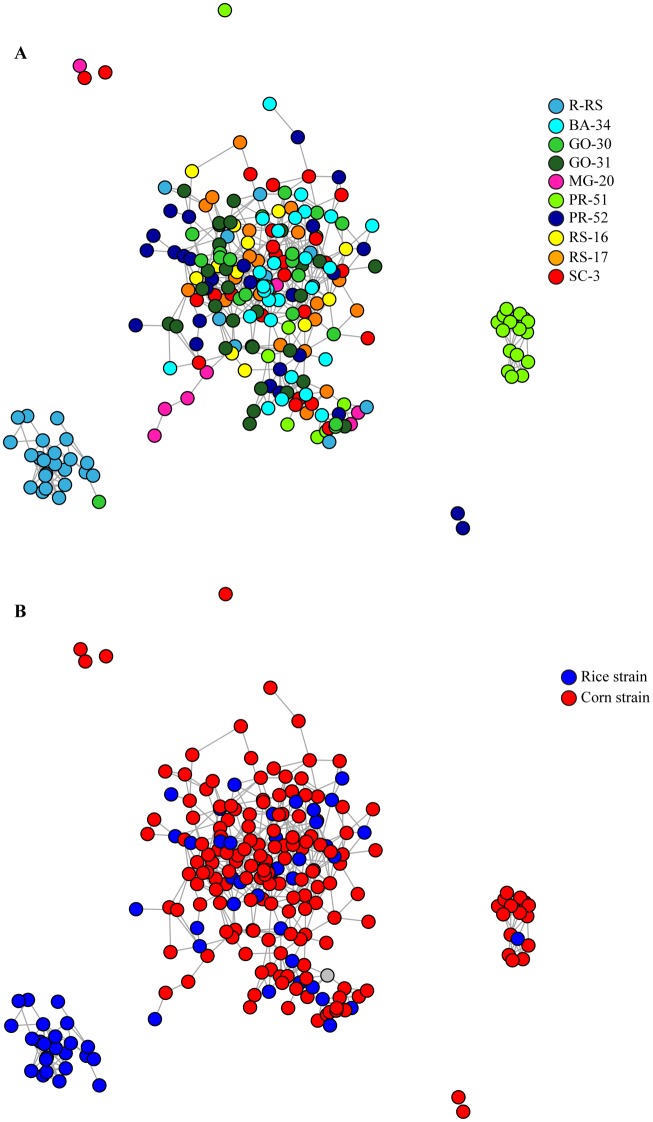
Network. Network of Brazilian populations of *S*. *frugiperda* (FAW) at *k* = 10 with minimum-spanning tree (MST), based on 7664 SNPs. A) Colors represent each population sampled in the field; B) Colors represent corn- (red) and rice- (blue) strains of FAW.

The network-based approach to estimate population structure indicated that most of the individuals collected from rice (R-RS) (in light blue in Fig 5A) are isolated from the remaining populations, and even when the nearest-neighbor parameter *k* was increased to 40 there was no connection with the main network. Curiously, only one other individual, from population GO-30 (rice strain) was connected with the individuals from R-RS. Sixteen individuals from PR-51 (from a total of 28) formed a second isolated network (in light green in Fig 5A), and this network is connected with the main arrangement when *k* = 40. Individuals from all other populations compose the main network, with a few individuals from different populations unconnected. The network structure obtained when corn and rice strains were considered make it evident that rice-strain individuals from R-RS are isolated, as are corn-strain individuals from PR-51, while all other individuals are connected in the main network, regardless of their strain (Fig 5B).

**Fig 6:**
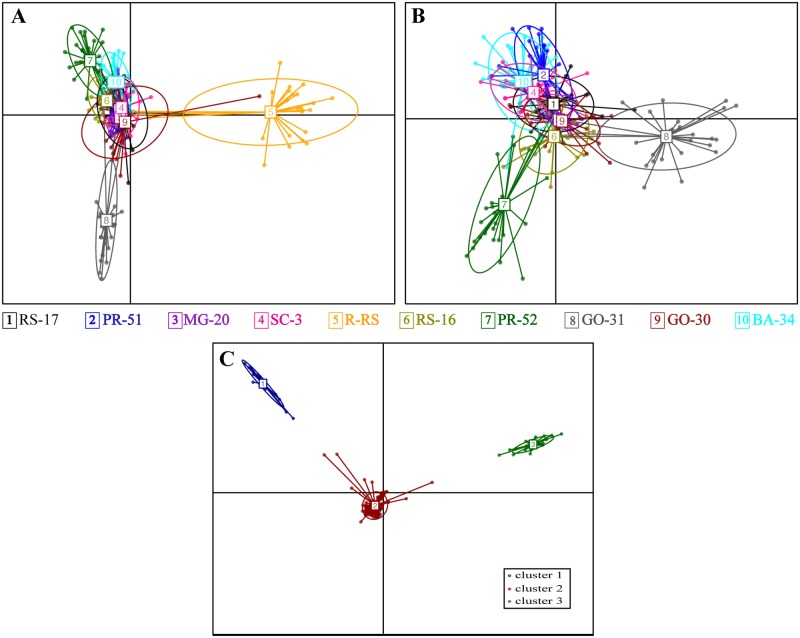
DAPC scatterplots. DAPC scatterplots, with the individuals represented as dots and the groups as inertia ellipses. A) Relationships among individuals of *S*. *frugiperda* (FAW) collected from corn and rice fields; B) Relationships among individuals of FAW collected from corn. Each color represents a population; C) Optimal number of clusters for Brazilian populations of *S*. *frugiperda* identified using *k*-means in adegenet, including samples from corn and rice fields.

The DAPC including all populations discriminated population R-RS on axis X and population GO-31 on axis Y (Fig 6A). When we removed population R-RS, collected from rice from the analysis, we discriminated population GO-31 on axis X and population PR-52 on axis Y (Fig 6B). The find-clusters analysis of DAPC resulted in three clusters (Fig 6C). Cluster 1 is composed of 15 of 28 individuals from PR-51; and cluster 2 is composed of the remaining individuals from PR-51, a few individuals from R-RS and all individuals from the other localities sampled. Cluster 3 is comprised of 23 of 27 individuals from R-RS and one individual from GO-30. When we removed R-RS, the find-clusters analysis resulted in two clusters, corresponding to clusters 1 and 2 found previously, with the individual from GO-30 in cluster 2.

Fifty loci were above the threshold of 0.0008 fixed to estimate the contributions of alleles to the clustering pattern (S4 Fig). All but three of these alleles are putatively under selection according to our previous analysis; these three loci are below the 99% confidence interval that we set for the Lositan analyses, but are close to a 90% confidence interval. Thirty of these alleles are fixed in cluster 3. Thirteen loci were annotated in the transcriptome of FAW (S3 Table). Descriptions of these loci include enzymes involved in oxidoreductase activity, such as malate dehydrogenase and sepiapterin reductase, both fixed in cluster 3, and in transferase activity, such as speckle targeted PIP5K1A-regulated poly(A) polymerase and ephrin type-B receptor 1-B. Two loci were suggested to be involved in the carbohydrate metabolic process, malate dehydrogenase and alpha-mannosidase 2, and one locus is related to *Bt*-resistance in insects, cadherin-related tumor suppressor (locus 14975). This locus was mapped to the contig 54090 in the transcriptome of *S*. *frugiperda*, a contig with 351 amino acids. When contig 54090 was blasted against the NCBI data bank, two sequences were indicated with the highest similarity, a predicted *Spodoptera litura* cadherin-related tumor suppressor (LOC111348058) (identities 984/1054, 93%), and a predicted *Helicoverpa armigera* cadherin-related tumor suppressor (LOC110371604) (identities 855/1048, 81%). This contig showed the highest similarity (98%) with the scaffold 46658 when blasted against the corn variant assembly 3.1 of the genome of *S*. *frugiperda*, and with the SFRU RICE 028070 when blasted against the rice variant assembly 1.0 (99%). The alignment of the contig 54090 with several cadherin available showed a high similarity to the cadherin repeats 10–12 involved in the interaction with Cry proteins in other lepidopterans (S5 Fig).

## Discussion

### Strain identification

It has long been recognized that host plant-related strains of the FAW *Spodoptera frugiperda* show preferences for specific host plants. We confirmed the expectation of host preference of FAW strains for Brazilian populations, through the strain identification of field-collected individuals. Populations collected from corn were composed primarily of corn-strain individuals, with a varied low percentage of rice-strain individuals; while the population collected from rice was comprised almost entirely of rice-strain individuals. Even with only one population sampled from rice, this is the first time that this pattern has been described for the entire Brazilian distribution of FAW. The predominance of rice-strain insects on rice and a mixture of corn- and rice-strain insects on corn was also found for other populations from Rio Grande do Sul, Brazil [15, 92]; from Tolima, Colombia [93]; from North and Central America [54], and from Argentina [94]. This pattern, however, is not universal, and Juárez and collaborators [95], for instance, found that 100% of individuals collected on corn in Rio Grande do Sul, as well as in other localities, belonged to the rice strain.

Usually, the preference for a host plant in generalist insects is related either to differential larval survival in each plant or female oviposition preference and specificity for the host substrate. Differential survival may be related to mechanical barriers that can function to prevent corn-strain FAW larvae from feeding on rice, such as the silica content in rice leaves, since silica is a feeding deterrent for *S*. *frugiperda* larvae, mainly in the early stages of development [96, 97]. Busato and collaborators [98] suggested that the preference for corn is due to the presence of high amounts of silica in rice leaves, which can interfere with insect digestion. However, both rice and corn contain silica in their leaves, although it is not known if the varieties of these plants in Brazil contain the same amounts of silica. In addition, rice strain larvae also show preference for other non-rice grasses to corn [38], and other explanations besides the mechanical hypothesis need to be addressed.

Secondary metabolites on corn and rice plants could also function as chemical barriers to the free feeding of corn-strain larvae on rice. Aerial parts of corn contain the benzoxazinoid (BXD) hydroxamic acid, DIMBOA (2,4-dihydroxy-7-methoxy-1,4-benzoxazin-3-one), which confers resistance to herbivorous insects and pathogens because of its antifeeding, insecticidal, antimicrobial and allelopathic properties [99–101]. Remarkably, although DIMBOA has a feeding deterrent and/or toxic action on several lepidopteran pest species [99, 102], it acts as a feeding stimulant for *S*. *frugiperda* and enhances FAW larval growth at low concentrations [100, 103]. This compound, however, is absent in rice, and its absence may make rice less attractive to corn-strain larvae. Molecular-dating analyses suggest that the two strains of the FAW may have diverged more than 2 My ago [104], that is, before the domestication or introduction of corn and rice in the Western Hemisphere, and there is no information on which is the ancestral host plant for this insect. It is impossible, therefore, to determine if the presence of DIMBOA in corn, or its absence in rice, constitutes a selective pressure on the two FAW strains.

Other plant secondary compounds may also be responsible for the FAW differential use of host plants, such as phytoecdysteroids. Phytoecdysteroids are steroidal compounds synthesized by plants that interfere with insect ecdysis and development [105, 106]. They are analogs to insect molting hormones, and are also believed to function as feeding deterrents against attack by non-adapted phytophagous insects [105]. In insects, ecdysone oxidase is an enzyme that breaks down excess ecdysteroids after recurrent molts [107]. In our study, locus 4448 was annotated as a GO related to ecdysone oxidase activity. This locus is associated with the host plant, showing opposite allele frequencies in individuals collected from corn or rice, and is also under selection in our analyses. A putative ecdysone oxidase protein was also described as down-regulated in the salivary proteome of the FAW corn-strain larvae [89]. Significant differences in allele frequencies in individuals collected from corn and rice at a locus annotated as ecdysone oxidase suggest that insects feeding on each host can be under divergent selective pressure from plant phytoecdysteroids. If this is the case, rice-strain larvae, which are preferentially found on rice, may currently show adaptive differences in relation to corn-strain larvae as a response to these compounds. In fact, phytoecdysteroids were found in rice but not in corn [108, 109], although corn proved to be able to convert cholesterol to a 20-hydroxyecdysone conjugate [110]. Together, our results and those of [89] suggest that ecdysone oxidase shows a variable response related to the FAW strain and/or the host plant where the larvae are feeding.

On the other hand, female oviposition preference was suggested for FAW based on host plants choice trials at laboratory conditions [111–113]. A mixed pattern rises when all studies are considered, but in general rice strain females show a stronger preference towards grasses as an ovipositional substrate, while corn strain females show a mostly indiscriminate pattern. Several behavioral and ecological reasons may be responsible for this mixed pattern in corn strain females, including higher sensitivity to experimental condition and/or colony age [111].

Several possible pre- and poszygotic isolation barriers have been postulated to explain the asymmetric use of host plants by *S*. *frugiperda* larvae, such as variation in the competitive abilities of the two strains, behavioral isolation through sexual communication mediated by different pheromone blends, and differential timing of reproduction. In conclusion, each barrier could theoretically contribute individually and simultaneously to the host-plant preference in FAW larvae [39].

The main question, however, is whether individuals of the two strains are reproductively isolated in the field, and the answer we are able to provide at this moment is "partially". Most of the rice-strain individuals collected from rice were genetically isolated from corn-strain individuals in general, even if they were geographically close, but the rice-strain individuals collected from corn belonged to the same genetic cluster as their corn-strain co-host fellows. The marker that we used to identify FAW strains may be responsible for this pattern. Here we applied the most often used strain-specific *Msp*I site in the mtDNA COI region to characterize strain genotypes of FAW [114], and we recognize that other markers have shown discrepancies with mtDNA in FAW strain identification [50, 51, 54, 115]. Although all studies have indicated high levels of success of the mitochondrial marker in the identification of FAW strains, it is unable to identify corn-rice hybrids. In fact, when markers capable of detecting hybrids in FAW populations were investigated, they indicated a greater presence of potential hybrids on corn compared to rice plants [54]. It is in fact expected, considering that RS individuals are usually found on corn, but the CS individuals are less commonly found using rice. The presence of hybrids is evidence of cross-strain reproduction on corn, and indicates that individuals on corn, irrespective of strain, are genetically more similar to each other. In contrast, if for any reason corn-strain individuals are less capable of feeding on rice, only pure rice-strain individuals would be found on rice, and these individuals are less similar genetically in comparison to those from corn. We believe that the isolated networks (Fig 5) are indication of hybrids. The isolation of the individuals sampled on rice from the others from the same population is indicative of reproductive isolation, and in fact they should be pure-rice strain. While individuals sampled from the same rice population found among corn strain individuals should be rice-corn hybrids since they keep same interbreeding with corn strain individuals, but are characterized as rice strain when the mitochondrial marker is evaluated. Polymorphisms in the Triosylphosphate isomerase gene (*Tpi*) are capable to distinguish between the two FAW strains, and to indicate corn-rice hybrids [115]. We did not test *Tpi* here, and we did not find it in the loci obtained by genotyping-by-sequencing technique, so we do not have the hybrid information for the populations of FAW we studied.

The fact that several loci responsible for the genetic cluster configuration in the DAPC analysis are fixed in the rice cluster (cluster 3) consists an additional evidence that these rice-strain individuals are genetically isolated from the other Brazilian populations of FAW. As these loci include molecular functions such as the carbohydrate metabolic process and oxidoreductase activity, both functions related to digestion in insects, they also suggest the existence of adaptive differences in the rice-strain individuals that allow them to feed on rice.

### Genetic structure

Pairs of populations of polyphagous herbivorous insects using different host plants are expected to be more reproductively isolated than pairs using the same host plant if they are under selective pressure due to ecological-speciation mechanisms. Here we were able to show two important results related to this expectation: 1) the only population of *S*. *frugiperda* collected from rice is more isolated from all other populations collected from corn, even from geographically close populations; and 2) all populations collected from corn show low genetic structure compared to each other. Some individuals collected from rice, however, are connected to individuals from corn, which indicates current gene flow among individuals of FAW feeding on these different host plants. Our results coincide with studies that found the host-plant association as the main reason for the genetic differentiation within and among populations of *S*. *frugiperda* [49, 51], but also with studies that have suggested causes other than host-plant preference as responsible for the genetic distinctiveness of the FAW populations [50, 116].

If genetic structure is at least partially due to host-plant association, corn and rice populations could be under different selective pressures. Irrespective of the strain to which an individual belongs, if it is feeding on corn it is adapted to this host plant. So, how do these individuals differ from individuals that feed on rice in the field? Our association analyses indicated that loci significantly associated with the host plant where the individual was collected included unigenes related to digestion in insects, and to resistance to insecticides and genetically modified plants. Zinc carboxypeptidases, for instance, are related to *Bt*-resistance in Lepidoptera [71–73], and they were purified from larval guts of the corn earworm *Helicoverpa armigera* [117]. Zinc carboxypeptidase was also present in the list of gut genes with expression differences between susceptible and resistant larvae of *Ostrinia nubilalis* fed on transgenic *Cry1Ab* or non-transgenic corn for 6 hours [118]. *Bt*-corn seeds with Cry toxins were introduced to reduce FAW infestations in Brazil in 2008 [36], and in addition to insecticides, FAW can also develop resistance to *Bt*-crops. It is interesting that larvae found in this study feeding on non-*Bt*-corn and on non-transgenic rice show different allele frequencies at this locus, which indicates that these individuals are under divergent selective pressures on this trait.

Cadherin, also associated with resistance to *Bt*-crops [71–73], is related to *Cry1A* toxin-binding in lepidopteran insects [119] and is the most frequent mechanism of resistance to *Bt* Cry toxins, due to changes in receptor binding, as reported for *H*. *armigera* [120]. The locus 8953 annotated as cadherin is not associated with the host plant, but it is significantly associated with the strain, although we were not able to infer its similarity with cadherin regions involved in the interaction with Cry proteins. As the differentiation of FAW strains is intimately associated with the host-plant preference, differences in allele frequency at this locus may also be a consequence of different pressures of *Bt*-corn and non-transgenic rice on this trait. The locus 14975, also annotated as a cadherin domain, is a locus that contributed to the arrangement of individuals in three clusters. This locus showed a high similarity with sequences of cadherin well characterized in other pest species of Lepidoptera, such as *Helicoverpa armigera* [121] and *Manduca sexta* [122], including the domains 10–12 involved in the interaction with Cry proteins. That can be considered as evidence that population genetic structure in *S*. *frugiperda* is also shaped by the response of its populations to the methods of control used at field conditions.

Two other loci were significantly associated with both host plant and strain in the FAW populations: a locus annotated as glutathione transferase showed a large difference in allele frequency in corn and rice plants and strains. Glutathione transferases are closely associated with insecticide resistance in insects, including pyrethroids [90, 123], intensively applied to control FAW populations in corn, and resistance to pyrethroids has been described for Brazilian populations [124, 125].

Another locus associated with both features was annotated as insulin receptor, which was shown to play an essential role in feeding behavior in insects, as a key metabolic hormone related to carbohydrate and lipid metabolism [91]. In the comparison between CS and RS genomes, an insulin-like peptide was shown to be under positive selective pressure [52].

All those annotated loci simultaneously associated with the host plant and strain are also putatively under selection in the populations of FAW we investigated, but we found other loci under selection that are also related to resistance to *Bt*-crops and insecticides. In addition to the unigene annotated as zinc carboxypeptidase, we found two loci annotated as ABC transporter, another mechanism related to *Bt*-resistance [71–73], and both loci showed a high similarity with ABC genes associated to *Bt* resistance in other species of Lepidoptera. Furthermore, we found several unigenes annotated as cytochrome P450 and carboxylesterases that, together with glutathione transferase, are related to increased detoxification of insecticides in insects [74]. Our analyses also indicated loci under selection in the field population of FAW that are also under selective pressure in the comparative analyses of CS and RS genomes: alanine aminotransferase and phosphomannomutase (both involved in digestion and metabolism in insects), and chitin binding, related to the gut peritrophic membrane [52]. As we inferred that loci putatively under selection are the major factor responsible for the genetic structure of FAW populations, we can assume that loci annotated as members of important gene families related to resistance to pest control are among the loci responsible for this structuring in the field.

A recurrent question related to FAW is its taxonomic status: are the two host-related strains sibling species or races? Genome-wide analyses have indeed found significant genomic differentiation between the two strains [52]. Several terms have been used to describe the FAW strains, occasionally used as synonyms in the same manuscript, such as host strains [38, 39, 41, 42, 48, 53, 54, 95], host races [39], host assemblages [54], host forms [50, 53], biotypes [49], ecological races [53], genetically differentiated forms [54], genetic groups [39], and sibling species [38, 53]. Here, we applied the most frequently used designation, host strain. Our results agree with this label, since populations with individuals of both strains maintain gene flow, although they show enough dissimilarities to identify an ongoing process of differentiation.

## Conclusions

Considering our initial questions, there is evidence that Brazilian populations of *S*. *frugiperda* are structured according to the host plant where they were collected, although we were able to sample only one population from rice, and pairs of populations using the same host plant are more genetically similar than pairs using different host plants. Populations collected from corn are genetically more similar to other populations collected from the same host plant, which indicates current or historic gene flow among those populations. The only population collected from rice is more isolated from all other populations from corn, even from geographically close populations. Other factors, however, also contribute to the genetic structure of Brazilian populations of FAW (*vide* population PR-51 isolated from other corn-collected populations). Loci putatively under selection are the main factors responsible for the genetic structure of these populations, which indicates that adaptive selection on important traits, including response to control tactics, is acting in the genetic differentiation of FAW populations in Brazil.

## Supporting information

S1 Table Loci putatively under selection. Gene Ontology annotation (GO) and description of loci putatively under selection.
(PDF)

S2 Table Association analyses. Gene Ontology (GO) annotation and molecular function description of loci associated to the host plant where the individuals were collected, to the individual strain or to the two features. All annotated loci simultaneously associated to the two features are also putatively under selection. A_1_ and A_2_ = two alleles forms presented in each loci; F_A_1_ = frequency of allele A_1_ in rice or corn host plant or strain; Bonf = Bonferroni adjusted significance values of each allele frequency comparison in each feature.
(PDF)

S3 Table Annotation of loci responsible for cluster formation. Gene Ontology (GO) annotation and description of loci that contributed to the arrangement of individuals in three clusters found in the DAPC analysis. † Alleles fixed in cluster 3, composed of individuals from R-RS. ‡ Aspect: M = Molecular function, B = Biological process, C = Cellular component.
(PDF)

S1 Fig Alignment of contig 12636 with sequences of ABC proteins from the literature. Sequence *Spodoptera litura* ABCC1-like (KM453742) was the best match when BLAST was used (Identities 1477/1635, 90%); sequence *Spodoptera exigua* ABCC2 (KM068116) is discussed in [126]; sequence *Spodoptera exigua* ABCC3 (KF926101) discussed in [83]; sequence Chrysomela tremula ABCB1 (KX686490) is discussed in [86]; sequence *Helicoverpa armigera* ABCA2 (KP259911) is annotated as in [85]. TpM = transporter motif (in blue); ATP = ATP-binding domains (in red); region of overlap with locus 12150 (in green), with the amino acid related to the polymorphism in the position 896 of the alignment (amino acid Leucine in the contig 12636, and polymorphism C/TTG in the locus 12150). Figure generated in Geneious v. 10.2 (Biomatters).
(PDF)

S2 Fig Alignment of contig 19063 with sequences of ABC proteins from the literature. Sequence *Spodoptera litura* subfamily F member is a predicted ATP-binding protein (LOC111362986), and was the best match when BLAST was used (Identities 1857/1981, 93%); sequence *Spodoptera exigua* ABCC2 (KM068116) is discussed in [126]; sequence *Spodoptera exigua* ABCC3 (KF926101) discussed in [83]; sequence Chrysomela tremula ABCB1 (KX686490) is discussed in [86]; sequence *Helicoverpa armigera* ABCA2 (KP259911) is annotated as in [85]. TpM = transporter motif (in blue); ATP = ATP-binding domains (in red); region of overlap with locus 12150 (in green), with the amino acid related to the polymorphism in the position 896 of the alignment (amino acid Leucine in the contig 12636, and polymorphism C/TTG in the locus 12150). Figure generated in Geneious v. 10.2 (Biomatters).
(PDF)

S3 Fig Alignment of contig 63619 with sequences of cadherin proteins from the literature. Sequence *Helicoverpa armigera* (LOC 110371604) is a predicted cadherin-related tumor suppressor; sequence *Helicoverpa armigera* (BtR) (AY647974) is annotated as in [121], and additional information [127, 128]; sequence *Manduca sexta* (BT-R1) (AF319973) is annotated as in [122]. EC = cadherin repeats (in red); TBR = putative Cry1Ac toxin binding region (in blue). Figure generated in Geneious v. 10.2 (Biomatters).
(PDF)

S4 Fig Loci responsible for cluster formation. Loci contributions to clustering pattern above the threshold of 0.0008.
(PDF)

S5 Fig Alignment of contig 54090 with sequences of cadherin proteins from the literature. Sequence *Helicoverpa armigera* (LOC 110371604) is a predicted cadherin-related tumor suppressor; sequence *Helicoverpa armigera* (BtR) (AY647974) is annotated as in [121], and additional information [127, 128]; sequence *Manduca sexta* (BT-R1) (AF319973) is annotated as in [122]. EC = cadherin repeats (in red); TBR = putative Cry1Ac toxin binding region (in blue). Figure generated in Geneious v. 10.2 (Biomatters).
(PDF)
