# Supplementary material for: Loci under selection and markers associated with host plant and host-related strains shape the genetic structure of Brazilian populations of Spodoptera frugiperda (Lepidoptera, Noctuidae)
Source: PLoS One. 2018 May 22;13(5):e0197378. doi: 10.1371/journal.pone.0197378 (PMC5963752; doi:10.1371/journal.pone.0197378)
Supplement: S1 Table — Gene Ontology annotation (GO) and description of loci putatively under selection. (PDF) [file pone.0197378.s001.pdf]

Markers associated with host plant and host-related strains and the genetic structure of Brazilian populations of *Spodoptera frugiperda* (Lepidoptera, Noctuidae)

Karina Lucas Silva-Brandão, Aline Peruchi, Noemy Seraphim, Natália Faraj Murad, Renato Assis Carvalho, Juliano Ricardo Farias, Celso Omoto, Fernando Luis Cônsoli, Antonio Figueira, Marcelo Mendes Brandão

Supporting Information

S1 Table. Gene Ontology annotation (GO) and description of loci putatively under selection.

GO Description	GO_ID	Sequence Description
[acyl-carrier-protein] S-acetyltransferase activity	GO:0004313	fatty acid synthase (76193) PFAM: Thioesterase domain(PF00975.16:Thioesterase)
[acyl-carrier-protein] S-malonyltransferase activity	GO:0004314	fatty acid synthase (76193) PFAM: Thioesterase domain(PF00975.16:Thioesterase)
2-succinyl-6-hydroxy-2,4-cyclohexadiene-1-carboxylate synthase activity	GO:0070205	lipase 3-like (680683) peptidase: hypothetical protein (MER0424092:S33.A85) PFAM: alpha/beta hydrolase fold(PF00561.16:Abhydrolase_1)
3-hydroxypalmitoyl-[acyl-carrier-protein] dehydratase activity	GO:0004317	fatty acid synthase (76193) PFAM: Thioesterase domain(PF00975.16:Thioesterase)
3-oxoacyl-[acyl-carrier-protein] reductase (NADPH) activity	GO:0004316	fatty acid synthase (76193) PFAM: Thioesterase domain(PF00975.16:Thioesterase)
3-oxoacyl-[acyl-carrier-protein] synthase activity	GO:0004315	fatty acid synthase (76193) PFAM: Thioesterase domain(PF00975.16:Thioesterase)
3-oxoadipate enol-lactonase activity	GO:0047570	lipase 3-like (680683) peptidase: hypothetical protein (MER0424092:S33.A85) PFAM: alpha/beta hydrolase fold(PF00561.16:Abhydrolase_1)
3'-5' exonuclease activity	GO:0008408	DNA polymerase epsilon catalytic subunit A (66420) PFAM: Domain of unknown function (DUF1744)(PF08490.8:DUF1744)
4-aminobutyrate transaminase activity	GO:0003867	4-aminobutyrate aminotransferase, mitochondrial (76193) PFAM: Aminotransferase class-III(PF00202.17:Aminotran_3)
actin binding	GO:0003779	actin-binding LIM protein 3 (51655) PFAM: LIM domain(PF00412.18:LIM); myosin-VIIa (680683) PFAM: Myosin head (motor domain)(PF00063.17:Myosin_head)
acyl-[acyl-carrier-protein] hydrolase activity	GO:0016297	fatty acid synthase (76193) PFAM: Thioesterase domain(PF00975.16:Thioesterase)
alanine-tRNA ligase activity	GO:0004813	alanine--tRNA ligase, mitochondrial (680683)
alkaline phosphatase activity	GO:0004035	alkaline phosphatase-like (7091) PFAM: Alkaline phosphatase(PF00245.16:Alk_phosphatase)
alpha-mannosidase activity	GO:0004559	alpha-mannosidase 2-like (7091) PFAM: Glycosyl hydrolases family 38 C-terminal domain(PF07748.9:Glyco_hydro_38C)
amino acid transmembrane transporter activity	GO:0015171	cationic amino acid transporter 4 (680683) PFAM: Amino acid permease(PF13520.2:AA_permease_2)
aminoacyl-tRNA editing activity	GO:0002161	valine--tRNA ligase (7091) PFAM: Anticodon-binding domain of tRNA(PF08264.9:Anticodon_1)
aminoacyl-tRNA ligase activity	GO:0004812	valine--tRNA ligase (7091) PFAM: Anticodon-binding domain of tRNA(PF08264.9:Anticodon_1)
aminopeptidase activity	GO:0004177	probable aminopeptidase NPEPL1 (680683) peptidase: hypothetical protein KGM_11987 (MER0340753:M17.UPW) PFAM: Cytosol aminopeptidase family, catalytic domain(PF00883.17:Peptidase_M17)
anaphase-promoting complex binding	GO:0010997	fizzy-related protein homolog (7091)
antiporter activity	GO:0015297	sodium/hydrogen exchanger 8-like (51655) PFAM: Sodium/hydrogen exchanger family(PF00999.17:Na_H_Exchanger)
ARF guanyl-nucleotide exchange factor activity	GO:0005086	brefeldin A-inhibited guanine nucleotide-exchange protein 1 (76193) PFAM: Sec7 domain(PF01369.16:Sec7)
arginine kinase activity	GO:0004054	arginine kinase (66420)

aspartic-type endopeptidase activity	GO:0004190	uncharacterized LOC106141703 (680683)
ATP binding	GO:0005524	heat shock protein 83 (680683) PFAM: Hsp90 protein(PF00183.14:HSP90); dual specificity mitogen-activated protein kinase kinase 6 (7091) PFAM: Protein kinase domain(PF00069.21:Pkinase); insulin receptor (7091) PFAM: Furin-like cysteine rich region(PF00757.16:Furin-like)serine/threonine-protein kinase SIK2-like (680683) PFAM: Protein kinase domain(PF00069.21:Pkinase); epidermal growth factor receptor (76194) PFAM: Receptor L domain(PF01030.20:Recep_L_domain)
ATPase activity	GO:0016887	ATP-binding cassette sub-family F member 3 (680683) PFAM: ABC transporter(PF00005.23:ABC_tran)
ATPase activity, coupled to transmembrane movement of substances	GO:0042626	multidrug resistance-associated protein 1 (64793) PFAM: ABC transporter transmembrane region(PF00664.19:ABC_membrane)
AU-rich element binding	GO:0017091	exosome complex component RRP42 (680683) PFAM: 3' exoribonuclease family, domain 1(PF01138.17:RNase_PH)
beta-catenin binding	GO:0008013	adenomatous polyposis coli protein-like (66420)
calcium channel activity	GO:0005262	ryanodine receptor 44F (66420) PFAM: RIH domain(PF01365.17:RYDR_ITPR)
calcium ion binding	GO:0005509	basement membrane-specific heparan sulfate proteoglycan core protein (76193) inhibitor: PREDICTED: similar to ENSANGP00000022422 (MER0205277:I63.UPW) PFAM: Immunoglobulin I-set domain(PF07679.12:I-set)
calcium-dependent cysteine-type endopeptidase activity	GO:0004198	uncharacterized LOC106141840 (680683)
calmodulin binding	GO:0005516	probable phosphorylase b kinase regulatory subunit beta (76193)
carbohydrate binding	GO:0030246	alpha-mannosidase 2-like (7091) PFAM: Glycosyl hydrolases family 38 C-terminal domain(PF07748.9:Glyco_hydro_38C)
carboxy-lyase activity	GO:0016831	lipase 3-like (680683) peptidase: hypothetical protein (MER0424092:S33.A85) PFAM: alpha/beta hydrolase fold(PF00561.16:Abhydrolase_1)
carboxypeptidase activity	GO:0004180	retinoid-inducible serine carboxypeptidase-like (680683) peptidase: hypothetical protein KGM_01563 (MER0350452:S10.UPW) PFAM: Serine carboxypeptidase(PF00450.18:Peptidase_S10)
catalytic activity	GO:0003824	glutamine synthetase 1, mitochondrial (680683) PFAM: Niemann-Pick C1 N terminus(PF16414.1:NPC1_N); alanine aminotransferase 1 (7091) peptidase: GF17085 (MER0208656:S09.941) PFAM: Phospholipase/Carboxylesterase(PF02230.12:Abhydrolase_2)
cation binding	GO:0043169	alanine aminotransferase 1 (7091) peptidase: GF17085 (MER0208656:S09.941) PFAM: Phospholipase/Carboxylesterase(PF02230.12:Abhydrolase_2)
cation:chloride symporter activity	GO:0015377	solute carrier family 12 member 6 (7091) PFAM: Solute carrier family 12(PF03522.11:SLC12)
chitin binding	GO:0008061	putative uncharacterized protein DDB_G0291608 (680683)
cholinesterase activity	GO:0004104	alanine aminotransferase 1 (7091) peptidase: GF17085 (MER0208656:S09.941) PFAM: Phospholipase/Carboxylesterase(PF02230.12:Abhydrolase_2)
copper ion binding	GO:0005507	laccase-4-like (680683) PFAM: Multicopper oxidase(PF07732.11:Cu-oxidase_3)
copper ion transmembrane transporter activity	GO:0005375	copper transporter (7091) PFAM: Ctr copper transporter family(PF04145.11:Ctr)
cysteine-type peptidase activity	GO:0008234	uncharacterized peptidase C1-like protein F26E4.3 (66420) peptidase: tubulointerstitial nephritis antigen (MER0345265:C01.UPW) PFAM: Papain family cysteine protease(PF00112.19:Peptidase_C1)
cytochrome-c oxidase activity	GO:0004129	cytochrome c oxidase subunit 6B1 (7091)
deaminase activity	GO:0019239	adenosine deaminase CECR1-like (680683) PFAM: Adenosine/AMP deaminase(PF00962.18:A_deaminase)
diacylglycerol kinase activity	GO:0004143	diacylglycerol kinase theta (66420) PFAM: Phorbol esters/diacylglycerol binding domain (C1 domain)(PF00130.18:C1_1)
dihydrolipoyllysine-residue succinyltransferase activity	GO:0004149	dihydrolipoyllysine-residue succinyltransferase component of 2-oxoglutarate dehydrogenase complex, mitochondrial (7091) PFAM: 2-oxoacid dehydrogenases acyltransferase (catalytic domain)(PF00198.19:2-oxoacid_dh)

DNA binding	GO:0003677	homeobox protein Hox-A1-like (76193) PFAM: Homeobox domain(PF00046.25:Homeobox); sex determination protein fruitless-like (7091) PFAM: BTB/POZ domain(PF00651.27:BTB)
DNA-directed DNA polymerase activity	GO:0003887	DNA polymerase epsilon catalytic subunit A (66420) PFAM: Domain of unknown function (DUF1744)(PF08490.8:DUF1744)
ecdysone oxidase activity	GO:0047875	ecdysone oxidase (7091) PFAM: GMC oxidoreductase(PF00732.15:GMC_oxred_N)
endo-1,4-beta-xylanase activity	GO:0031176	alanine aminotransferase 1 (7091) peptidase: GF17085 (MER0208656:S09.941) PFAM: Phospholipase/Carboxylesterase(PF02230.12:Abhydrolase_2)
endonuclease activity	GO:0004519	uncharacterized LOC106108224 (76194) PFAM: Endonuclease-reverse transcriptase (PF14529.2:Exo_endo_phos_2)
endopeptidase inhibitor activity	GO:0004866	alpha-2-macroglobulin-like (7091) inhibitor: alpha 2-macroglobulin (MER0177021:I39.UPW) PFAM: A-macroglobulin complement component(PF07678.10:A2M_comp)
enoyl-[acyl-carrier-protein] reductase (NADPH, B-specific) activity	GO:0004319	fatty acid synthase (76193) PFAM: Thioesterase domain(PF00975.16:Thioesterase)
ephrin receptor activity	GO:0005003	ephrin type-B receptor 1-B (51655) PFAM: Ephrin receptor ligand binding domain(PF01404.15:Ephrin_lbd)
extracellular matrix binding	GO:0050840	SPARC-related modular calcium-binding protein 1 (76193) inhibitor: AGAP007489-PA (MER0023751:I31.UPW) PFAM: Thyroglobulin type-1 repeat(PF00086.14:Thyroglobulin_1)
fatty acid synthase activity	GO:0004312	fatty acid synthase (76193) PFAM: Thioesterase domain(PF00975.16:Thioesterase)
fatty-acyl-CoA reductase (alcohol-forming) activity	GO:0080019	putative fatty acyl-CoA reductase CG8306 (7091) PFAM: Male sterility protein(PF03015.15:Sterile)
flavin adenine dinucleotide binding	GO:0050660	glucose dehydrogenase [FAD, quinone]-like (680683); synaptic vesicle glycoprotein 2A-like (680683) PFAM: Sugar (and other) transporter(PF00083.20:Sugar_tr); ecdysone oxidase (7091) PFAM: GMC oxidoreductase(PF00732.15:GMC_oxred_N)
fucosyltransferase activity	GO:0008417	alpha-(1,3)-fucosyltransferase 10-like (66420) PFAM: Glycosyltransferase family 10 (fucosyltransferase) C-term(PF00852.15:Glyco_transf_10)
G-protein coupled receptor activity	GO:0004930	basement membrane-specific heparan sulfate proteoglycan core protein (76193) inhibitor: PREDICTED: similar to ENSANGP00000022422 (MER0205277:I63.UPW) PFAM: Immunoglobulin I-set domain(PF07679.12:I-set)
glucosamine-6-phosphate deaminase activity	GO:0004342	glucosamine-6-phosphate isomerase (680683) PFAM: Glucosamine-6-phosphate isomerases/6-phosphogluconolactonase(PF01182.16:Glucosamine_iso)
glucose oxidase activity	GO:0046562	glucose dehydrogenase [FAD, quinone]-like (680683)
glucuronosyltransferase activity	GO:0015020	PFAM: UDP-glucoronosyl and UDP-glucosyl transferase(PF00201.14:UDPGT)
glutamate-ammonia ligase activity	GO:0004356	glutamine synthetase 1, mitochondrial (680683) PFAM: Niemann-Pick C1 N terminus(PF16414.1:NPC1_N)
glutathione transferase activity	GO:0004364	glutathione S-transferase sigma 1 (7091)
glycogen phosphorylase activity	GO:0008184	glycogen phosphorylase (76193) PFAM: Carbohydrate phosphorylase(PF00343.16:Phosphorylase)
GTP binding	GO:0005525	elongation factor 1-alpha 2 (7091) PFAM: Elongation factor Tu GTP binding domain(PF00009.23:GTP_EFTU)
GTPase activator activity	GO:0005096	rho GTPase-activating protein 21-A-like (680683)
GTPase activity	GO:0003924	elongation factor 1-alpha 2 (7091) PFAM: Elongation factor Tu GTP binding domain(PF00009.23:GTP_EFTU)
guanylate cyclase activity	GO:0004383	uncharacterized LOC101744003 (7091)
haloacetate dehalogenase activity	GO:0018785	lipase 3-like (680683) peptidase: hypothetical protein (MER0424092:S33.A85) PFAM: alpha/beta hydrolase fold(PF00561.16:Abhydrolase_1)
helicase activity	GO:0004386	putative U5 small nuclear ribonucleoprotein 200 kDa helicase (680683) PFAM: Sec63 Brl domain(PF02889.12:Sec63)
heme binding	GO:0020037	PFAM: Cytochrome P450(PF00067.18:p450); cytochrome P450 6B7-like (680683)
heparan sulfate proteoglycan binding	GO:0043395	glypican-6 (76194) PFAM: Glypican(PF01153.15:Glypican)

hydrolase activity	GO:0016787	myrosinase 1-like (76194) PFAM: Glycosyl hydrolase family 1(PF00232.14:Glyco_hydro_1)
hydrolase activity, acting on ester bonds	GO:0016788	fatty acid synthase (76193) PFAM: Thioesterase domain(PF00975.16:Thioesterase)
hydrolase activity, hydrolyzing O-glycosyl compounds	GO:0004553	myrosinase 1-like (76194) PFAM: Glycosyl hydrolase family 1(PF00232.14:Glyco_hydro_1)
hydroxymethyl-, formyl- and related transferase activity	GO:0016742	trifunctional purine biosynthetic protein adenosine-3 (680683) PFAM: Formyl transferase(PF00551.15:Formyl_trans_N)
inositol-3-phosphate synthase activity	GO:0004512	inositol-3-phosphate synthase (680683) PFAM: Myo-inositol-1-phosphate synthase(PF07994.8:NAD_binding_5)
insulin receptor substrate binding	GO:0043560	insulin receptor (7091) PFAM: Furin-like cysteine rich region(PF00757.16:Furin-like)
intramolecular transferase activity, phosphotransferases	GO:0016868	phosphoglucomutase (680683) PFAM: Phosphoglucomutase/phosphomannomutase, alpha/beta/alpha domain III(PF02880.12:PGM_PMM_III)
ion channel activity	GO:0005216	ryanodine receptor 44F (66420) PFAM: RIH domain(PF01365.17:RYDR_ITPR)
ionotropic glutamate receptor activity	GO:0004970	UNKNOWN ANNOTATION
iron ion binding	GO:0005506	PFAM: Cytochrome P450(PF00067.18:p450); cytochrome P450 6B7-like (680683)
isomerase activity	GO:0016853	glucosamine-6-phosphate isomerase (680683) PFAM: Glucosamine-6-phosphate isomerases/6-phosphogluconolactonase(PF01182.16:Glucosamine_iso)
kinase activity	GO:0016301	dual specificity mitogen-activated protein kinase kinase 6 (7091) PFAM: Protein kinase domain(PF00069.21:Pkinase); serine/threonine-protein kinase SIK2-like (680683)
kinesin binding	GO:0019894	kinesin-associated protein 3 (7091) PFAM: Kinesin-associated protein (KAP)(PF05804.8:KAP)
L-amino acid transmembrane transporter activity	GO:0015179	cationic amino acid transporter 4 (680683) PFAM: Amino acid permease(PF13520.2:AA_permease_2)
ligase activity	GO:0016874	glutamine synthetase 1, mitochondrial (680683) PFAM: Niemann-Pick C1 N terminus(PF16414.1:NPC1_N)
ligase activity, forming aminoacyl-tRNA and related compounds	GO:0016876	alanine--tRNA ligase, mitochondrial (680683)
lyase activity	GO:0016829	enolase-like (680683) PFAM: Enolase, C-terminal TIM barrel domain(PF00113.18:Enolase_C)
magnesium chelatase activity	GO:0016851	lipase 3-like (680683) peptidase: hypothetical protein (MER0424092:S33.A85) PFAM: alpha/beta hydrolase fold(PF00561.16:Abhydrolase_1)
magnesium ion binding	GO:0000287	enolase-like (680683) PFAM: Enolase, C-terminal TIM barrel domain(PF00113.18:Enolase_C)
mannosidase activity	GO:0015923	alpha-mannosidase 2-like (7091) PFAM: Glycosyl hydrolases family 38 C-terminal domain(PF07748.9:Glyco_hydro_38C)
metal ion binding	GO:0046872	transferrin-like (51655) peptidase: Transferrin (MER0015660:S60.UPW) PFAM: Transferrin(PF00405.13:Transferrin); cytochrome P450 6B7-like (680683) PFAM: Cytochrome P450(PF00067.18:p450) sorbitol dehydrogenase (7091) PFAM: Alcohol dehydrogenase GroES-like domain(PF08240.8:ADH_N); sex determination protein fruitless-like (7091) PFAM: BTB/POZ domain(PF00651.27:BTB)
metal ion transmembrane transporter activity	GO:0046873	zinc transporter ZIP1-like (680683) PFAM: ZIP Zinc transporter(PF02535.18:Zip)
metallocarboxypeptidase activity	GO:0004181	carboxypeptidase B-like (76194) peptidase: ebiP7330 (MER0019052:M14.A18) PFAM: Zinc carboxypeptidase(PF00246.20:Peptidase_M14)
metalloexopeptidase activity	GO:0008235	probable aminopeptidase NPEPL1 (680683) peptidase: hypothetical protein KGM_11987 (MER0340753:M17.UPW) PFAM: Cytosol aminopeptidase family, catalytic domain(PF00883.17:Peptidase_M17)
methyltransferase activity	GO:0008168	lysine-specific demethylase 4C-like (680683) PFAM: JmjC domain, hydroxylase(PF02373.18:JmjC)
MHC class I receptor activity	GO:0032393	basement membrane-specific heparan sulfate proteoglycan core protein (76193) inhibitor: PREDICTED: similar to ENSANGP00000022422 (MER0205277:I63.UPW) PFAM: Immunoglobulin I-set domain(PF07679.12:I-set)
microtubule binding	GO:0008017	chromosome-associated kinesin KIF4-like (66420)

microtubule motor activity	GO:0003777	dynein heavy chain 3, axonemal (66420) PFAM: Dynein heavy chain and region D6 of dynein motor(PF03028.11:Dynein_heavy)
monooxygenase activity	GO:0004497	PFAM: Cytochrome P450(PF00067.18:p450); cytochrome P450 6B7-like (680683)
motor activity	GO:0003774	unconventional myosin-XVIIIa (680683) PFAM: Myosin head (motor domain)(PF00063.17:Myosin_head)
myristoyl-[acyl-carrier-protein] hydrolase activity	GO:0016295	fatty acid synthase (76193) PFAM: Thioesterase domain(PF00975.16:Thioesterase)
neuropeptide Y receptor activity	GO:0004983	orexin receptor type 1-like (680683)
nucleic acid binding	GO:0003676	zinc finger protein Elbow (7091); sex determination protein fruitless-like (7091) PFAM: BTB/POZ domain(PF00651.27:BTB)
nucleotide binding	GO:0000166	elongation factor 1-alpha 2 (7091) PFAM: Elongation factor Tu GTP binding domain (PF00009.23:GTP_EFTU); multidrug resistance-associated protein 1 (64793) PFAM: ABC transporter transmembrane region (PF00664.19:ABC_membrane); insulin receptor (7091) PFAM: Furin-like cysteine rich region(PF00757.16:Furin-like)
nucleotidyltransferase activity	GO:0016779	speckle targeted PIP5K1A-regulated poly(A) polymerase-like (680683)
O-methyltransferase activity	GO:0008171	uncharacterized LOC106116927 (66420)
oleoyl-[acyl-carrier-protein] hydrolase activity	GO:0004320	fatty acid synthase (76193) PFAM: Thioesterase domain(PF00975.16:Thioesterase)
organic phosphonate transmembrane-transporting ATPase activity	GO:0015416	ATP-binding cassette sub-family F member 3 (680683) PFAM: ABC transporter(PF00005.23:ABC_tran)
ornithine decarboxylase activity	GO:0004586	ornithine decarboxylase 1-like (680683) PFAM: Pyridoxal-dependent decarboxylase, pyridoxal binding domain(PF02784.12:Orn_Arg_deC_N)
oxidoreductase activity	GO:0016491	malate dehydrogenase, mitochondrial-like (7091) PFAM: lactate/malate dehydrogenase, NAD binding domain(PF00056.19:Ldh_1_N); cytochrome P450 6B7-like (680683) PFAM: Cytochrome P450(PF00067.18:p450)
oxidoreductase activity, acting on CH-OH group of donors	GO:0016614	glucose dehydrogenase [FAD, quinone]-like (680683); ecdysone oxidase (7091) PFAM: GMC oxidoreductase(PF00732.15:GMC_oxred_N)
oxidoreductase activity, acting on paired donors, with incorporation or reduction of molecular oxygen	GO:0016705	PFAM: Cytochrome P450(PF00067.18:p450); cytochrome P450 6B7-like (680683)
oxidoreductase activity, acting on the CH-OH group of donors, NAD or NADP as acceptor	GO:0016616	malate dehydrogenase, mitochondrial-like (7091) PFAM: lactate/malate dehydrogenase, NAD binding domain(PF00056.19:Ldh_1_N)
palmitoyl-(protein) hydrolase activity	GO:0008474	lipase 3-like (680683) peptidase: hypothetical protein (MER0424092:S33.A85) PFAM: alpha/beta hydrolase fold(PF00561.16:Abhydrolase_1)
palmitoyl-[acyl-carrier-protein] hydrolase activity	GO:0016296	fatty acid synthase (76193) PFAM: Thioesterase domain(PF00975.16:Thioesterase)
peptidase activity	GO:0008233	trypsin, alkaline B-like (66420) peptidase: trypsin (MER0164768:S01.UPA) PFAM: Trypsin(PF00089.22:Trypsin)
phosphatase activity	GO:0016791	tyrosine-protein phosphatase 69D (680683) PFAM: Protein-tyrosine phosphatase(PF00102.23:Y_phosphatase)
phosphatidylinositol 3-kinase binding	GO:0043548	insulin receptor (7091) PFAM: Furin-like cysteine rich region(PF00757.16:Furin-like)
phospholipase A2 activity	GO:0004623	lipase 3-like (680683) peptidase: hypothetical protein (MER0424092:S33.A85) PFAM: alpha/beta hydrolase fold(PF00561.16:Abhydrolase_1)
phosphopantetheine binding	GO:0031177	fatty acid synthase (76193) PFAM: Thioesterase domain(PF00975.16:Thioesterase)
phosphoprotein phosphatase activity	GO:0004721	tyrosine-protein phosphatase 69D (680683) PFAM: Protein-tyrosine phosphatase(PF00102.23:Y_phosphatase)
phosphopyruvate hydratase activity	GO:0004634	enolase-like (680683) PFAM: Enolase, C-terminal TIM barrel domain(PF00113.18:Enolase_C)
phosphoribosylamine-glycine ligase activity	GO:0004637	trifunctional purine biosynthetic protein adenosine-3 (680683) PFAM: Formyl transferase(PF00551.15:Formyl_trans_N)
phosphoribosylformylglycinamidine cyclo-ligase activity	GO:0004641	trifunctional purine biosynthetic protein adenosine-3 (680683) PFAM: Formyl transferase(PF00551.15:Formyl_trans_N)

phosphoribosylglycinamide formyltransferase activity	GO:0004644	trifunctional purine biosynthetic protein adenosine-3 (680683) PFAM: Formyl transferase(PF00551.15:Formyl_trans_N)
phosphorylase activity	GO:0004645	glycogen phosphorylase (76193) PFAM: Carbohydrate phosphorylase(PF00343.16:Phosphorylase)
phosphotransferase activity, for other substituted phosphate groups	GO:0016780	ethanolaminephosphotransferase 1-like (7091)
pigment binding	GO:0031409	apolipoprotein D-like (7091)
polysaccharide binding	GO:0030247	uncharacterized peptidase C1-like protein F26E4.3 (66420) peptidase: tubulointerstitial nephritis antigen (MER0345265:C01.UPW) PFAM: Papain family cysteine protease(PF00112.19:Peptidase_C1)
potassium:chloride symporter activity	GO:0015379	solute carrier family 12 member 6 (7091) PFAM: Solute carrier family 12(PF03522.11:SLC12)
protein C-terminal methylesterase activity	GO:0051722	lipase 3-like (680683) peptidase: hypothetical protein (MER0424092:S33.A85) PFAM: alpha/beta hydrolase fold(PF00561.16:Abhydrolase_1)
protein dimerization activity	GO:0046983	enolase-like (680683) PFAM: Enolase, C-terminal TIM barrel domain(PF00113.18:Enolase_C)
protein kinase activity	GO:0004672	dual specificity mitogen-activated protein kinase kinase 6 (7091) PFAM: Protein kinase domain(PF00069.21:Pkinase); serine/threonine-protein kinase SIK2-like (680683) PFAM: Protein kinase domain(PF00069.21:Pkinase); insulin receptor (7091) PFAM: Furin-like cysteine rich region(PF00757.16:Furin-like)
protein kinase binding	GO:0019901	translational activator GCN1 (7091) PFAM: Domain of unknown function (DUF3554)(PF12074.4:DUF3554)
protein kinase regulator activity	GO:0019887	translational activator GCN1 (7091) PFAM: Domain of unknown function (DUF3554)(PF12074.4:DUF3554)
protein serine/threonine kinase activity	GO:0004674	dual specificity mitogen-activated protein kinase kinase 6 (7091) PFAM: Protein kinase domain(PF00069.21:Pkinase)
protein transporter activity	GO:0008565	vacuolar protein sorting-associated protein 35 (76194)
protein tyrosine kinase activity	GO:0004713	tyrosine-protein kinase transmembrane receptor Ror-like (680683) PFAM: Protein tyrosine kinase(PF07714.13:Pkinase_Tyr); insulin receptor (7091) PFAM: Furin-like cysteine rich region(PF00757.16:Furin-like)
protein tyrosine phosphatase activity	GO:0004725	tyrosine-protein phosphatase 69D (680683) PFAM: Protein-tyrosine phosphatase(PF00102.23:Y_phosphatase)
protein-glutamine gamma-glutamyltransferase activity	GO:0003810	annulin-like (680683) peptidase: RecName: Full=Annulin; AltName: Full=Protein-glutamine gamma-glutamyltransferase; AltName: Full=Transglutaminase (MER0705744:C111.UPW) PFAM: Transglutaminase family(PF00868.16:Transglut_N)
pyridoxal phosphate binding	GO:0030170	alanine aminotransferase 1 (7091) peptidase: GF17085 (MER0208656:S09.941) PFAM: Phospholipase/Carboxylesterase(PF02230.12:Abhydrolase_2)
Ran GTPase binding	GO:0008536	exportin-1 (66420) PFAM: CRM1 C terminal(PF08767.7:CRM1_C)
Renilla-luciferin 2-monooxygenase activity	GO:0050248	lipase 3-like (680683) peptidase: hypothetical protein (MER0424092:S33.A85) PFAM: alpha/beta hydrolase fold(PF00561.16:Abhydrolase_1)
ribonucleoprotein complex binding	GO:0043021	ribosome biogenesis protein BOP1 homolog (51655)
ribosome binding	GO:0043022	translational activator GCN1 (7091) PFAM: Domain of unknown function (DUF3554)(PF12074.4:DUF3554)
RNA binding	GO:0003723	40S ribosomal protein S29 (680683) PFAM: Ribosomal protein S14p/S29e(PF00253.17:Ribosomal_S14)
RNA methyltransferase activity	GO:0008173	uncharacterized LOC106116927 (66420)
RNA transmembrane transporter activity	GO:0051033	sid-1-related gene3 (7091) PFAM: dsRNA-gated channel SID-1(PF13965.2:SID-1_RNA_chan)
RNA-directed DNA polymerase activity	GO:0003964	UNKNOWN ANNOTATION
RNA-DNA hybrid ribonuclease activity	GO:0004523	uncharacterized LOC105389466 (51655) PFAM: RNase H(PF00075.20:RNase_H)
ryanodine-sensitive calcium-release channel activity	GO:0005219	ryanodine receptor 44F (66420) PFAM: RIH domain(PF01365.17:RYDR_ITPR)

scavenger receptor activity	GO:0005044	uncharacterized peptidase C1-like protein F26E4.3 (66420) peptidase: tubulointerstitial nephritis antigen (MER0345265:C01.UPW) PFAM: Papain family cysteine protease(PF00112.19:Peptidase_C1)
sepiapterin reductase activity	GO:0004757	sepiapterin reductase (7091) PFAM: short chain dehydrogenase(PF00106.21:adh_short)
sequence-specific DNA binding	GO:0043565	homeobox protein Hox-A1-like (76193) PFAM: Homeobox domain(PF00046.25:Homeobox)
serine-type carboxypeptidase activity	GO:0004185	retinoid-inducible serine carboxypeptidase-like (680683) peptidase: hypothetical protein KGM_01563 (MER0350452:S10.UPW) PFAM: Serine carboxypeptidase(PF00450.18:Peptidase_S10)
serine-type endopeptidase activity	GO:0004252	trypsin, alkaline B-like (66420) peptidase: trypsin (MER0164768:S01.UPA) PFAM: Trypsin(PF00089.22:Trypsin)
serine-type peptidase activity	GO:0008236	collagenase-like (76193) peptidase: hypothetical protein (MER0604345:S01.UPA) PFAM: Trypsin(PF00089.22:Trypsin); venom protease-like (680683) peptidase: hemolymph proteinase 9 (MER0047706:S01.200) PFAM: Trypsin(PF00089.22:Trypsin)
signal transducer activity	GO:0004871	G-protein coupled receptor moody (680683) PFAM: 7 transmembrane receptor (rhodopsin family)(PF00001.17:7tm_1)
small protein activating enzyme activity	GO:0008641	ubiquitin-like modifier-activating enzyme 5 (680683) PFAM: ThiF family(PF00899.17:ThiF)
sodium:potassium-exchanging ATPase activity	GO:0005391	sodium/potassium-transporting ATPase subunit alpha-like (680683) PFAM: E1-E2 ATPase(PF00122.16:E1-E2_ATPase)
sodium:proton antiporter activity	GO:0015385	sodium/hydrogen exchanger 8-like (51655) PFAM: Sodium/hydrogen exchanger family(PF00999.17:Na_H_Exchanger)
solute:proton antiporter activity	GO:0015299	sodium/hydrogen exchanger 8-like (51655) PFAM: Sodium/hydrogen exchanger family(PF00999.17:Na_H_Exchanger)
structural constituent of cuticle	GO:0042302	cuticular protein RR-2 motif 63 (7091)
structural constituent of ribosome	GO:0003735	40S ribosomal protein S29 (680683) PFAM: Ribosomal protein S14p/S29e(PF00253.17:Ribosomal_S14)
structural molecule activity	GO:0005198	clathrin heavy chain (680683) PFAM: Region in Clathrin and VPS(PF00637.16:Clathrin)
thiol oxidase activity	GO:0016972	FAD-linked sulfhydryl oxidase ALR (7091) PFAM: Erv1 / Alr family(PF04777.9:Evr1_Alr)
thiol-dependent ubiquitinyl hydrolase activity	GO:0036459	ubiquitin carboxyl-terminal hydrolase 35 (680683) peptidase: hypothetical protein (MER0608825:C19.UPW) PFAM: Ubiquitin carboxyl-terminal hydrolase(PF00443.25:UCH)
transaminase activity	GO:0008483	alanine aminotransferase 1 (7091) peptidase: GF17085 (MER0208656:S09.941) PFAM: Phospholipase/Carboxylesterase(PF02230.12:Abhydrolase_2)
transferase activity	GO:0016740	glutathione S-transferase sigma 1 (7091); UDP-glucuronosyltransferase 2B10-like (76193) PFAM: UDP-glucoronosyl and UDP-glucosyl transferase(PF00201.14:UDPGT); insulin receptor (7091) PFAM: Furin-like cysteine rich region(PF00757.16:Furin-like)
transferase activity, transferring acyl groups	GO:0016746	dihydrolipoyllysine-residue succinyltransferase component of 2-oxoglutarate dehydrogenase complex, mitochondrial (7091) PFAM: 2-oxoacid dehydrogenases acyltransferase (catalytic domain)(PF00198.19:2-oxoacid_dh); PFAM: UDP-glucoronosyl and UDP-glucosyl transferase(PF00201.14:UDPGT)
transferase activity, transferring hexosyl groups	GO:0016758	UDP-glucuronosyltransferase 2B10-like (76193) PFAM: UDP-glucoronosyl and UDP-glucosyl transferase(PF00201.14:UDPGT)
transferase activity, transferring phosphorus-containing groups	GO:0016772	arginine kinase (66420); speckle targeted PIP5K1A-regulated poly(A) polymerase-like (7091) PFAM: RNA 2'-phosphotransferase, Tpt1 / KptA family(PF01885.12:PTS_2-RNA)
translation elongation factor activity	GO:0003746	elongation factor 1-alpha 2 (7091) PFAM: Elongation factor Tu GTP binding domain(PF00009.23:GTP_EFTU)
translation initiation factor activity	GO:0003743	eukaryotic translation initiation factor 4E transporter-like (7091) PFAM: Nucleocytoplasmic shuttling protein for mRNA cap-binding EIF4E(PF10477.5:EIF4E-T)
transmembrane receptor protein tyrosine kinase activity	GO:0004714	insulin receptor (7091) PFAM: Furin-like cysteine rich region(PF00757.16:Furin-like)

transmembrane signaling receptor activity	GO:0004888	G-protein coupled receptor Mth2-like (76194) PFAM: 7 transmembrane receptor (Secretin family)(PF00002.20:7tm_2)
transmembrane transporter activity	GO:0022857	facilitated trehalose transporter Tret1-like (680683) PFAM: Sugar (and other) transporter(PF00083.20:Sugar_tr)
transporter activity	GO:0005215	facilitated trehalose transporter Tret1-like (680683) PFAM: Sugar (and other) transporter(PF00083.20:Sugar_tr); alpha-tocopherol transfer protein-like (680683) PFAM: CRAL/TRIO domain(PF00650.16:CRAL_TRIO); multidrug resistance-associated protein 1 (64793) PFAM: ABC transporter transmembrane region(PF00664.19:ABC_membrane)
triglyceride lipase activity	GO:0004806	alanine aminotransferase 1 (7091) peptidase: GF17085 (MER0208656:S09.941) PFAM: Phospholipase/Carboxylesterase(PF02230.12:Abhydrolase_2)
tRNA threonylcarbamoyladenosine dehydratase	GO:0061503	ubiquitin-like modifier-activating enzyme 5 (680683) PFAM: ThiF family(PF00899.17:ThiF)
ubiquitin binding	GO:0043130	E3 ubiquitin-protein ligase UBR5 (680683)
ubiquitin protein ligase binding	GO:0031625	cullin-2 (680683) PFAM: Cullin family(PF00888.18:Cullin)
ubiquitin-protein transferase activator activity	GO:0097027	fizzy-related protein homolog (7091)
ubiquitin-protein transferase activity	GO:0004842	E3 ubiquitin-protein ligase TRIP12 (76193) PFAM: HECT-domain (ubiquitin-transferase)(PF00632.21:HECT)
UFM1 activating enzyme activity	GO:0071566	ubiquitin-like modifier-activating enzyme 5 (680683) PFAM: ThiF family(PF00899.17:ThiF)
unfolded protein binding	GO:0051082	heat shock protein 83 (680683) PFAM: Hsp90 protein(PF00183.14:HSP90)
valine-tRNA ligase activity	GO:0004832	valine--tRNA ligase (7091) PFAM: Anticodon-binding domain of tRNA(PF08264.9:Anticodon_1)
voltage-gated potassium channel activity	GO:0005249	potassium/sodium hyperpolarization-activated cyclic nucleotide-gated channel 2 (66420) PFAM: Ion transport protein N-terminal(PF08412.6:Ion_trans_N)
zinc ion binding	GO:0008270	sorbitol dehydrogenase (7091) PFAM: Alcohol dehydrogenase GroES-like domain(PF08240.8:ADH_N)
