# Supplementary material for: Loci under selection and markers associated with host plant and host-related strains shape the genetic structure of Brazilian populations of Spodoptera frugiperda (Lepidoptera, Noctuidae)
Source: PLoS One. 2018 May 22;13(5):e0197378. doi: 10.1371/journal.pone.0197378 (PMC5963752; doi:10.1371/journal.pone.0197378)
Supplement: S2 Table — Gene Ontology (GO) annotation and molecular function description of loci associated to the host plant where the individuals were collected, to the individual strain or to the two features. All annotated loci simultaneously associated to the two features are also putatively under selection. A1 and A2 = two alleles forms presented in each loci; F_A1 = frequency of allele A1 in rice or corn host plant or strain; Bonf = Bonferroni adjusted significance values of each allele frequency comparison in each feature. (PDF) [file pone.0197378.s002.pdf]

**Markers associated with host plant and host-related strains and the genetic structure of Brazilian populations of *Spodoptera frugiperda* (Lepidoptera, Noctuidae)**

Karina Lucas Silva-Brandão, Aline Peruchi, Noemy Seraphim, Natália Faraj Murad, Renato Assis Carvalho, Juliano Ricardo Farias, Celso Omoto, Fernando Luis Cônsoli, Antonio Figueira, Marcelo Mendes Brandão

## Supporting Information

**S2 Table. Gene Ontology (GO) annotation and molecular function description of loci associated to the host plant where the individuals were collected, to the individual strain or to the two features.** All annotated loci simultaneously associated to the two features are also putatively under selection. $A_1$ and $A_2$ = two alleles forms presented in each loci; F_$A_1$ = frequency of allele $A_1$ in rice or corn host plant or strain; Bonf = Bonferroni adjusted significance values of each allele frequency comparison in each feature.

| **HOST** | | | | | | | | |
|---|---|---|---|---|---|---|---|---|
| Locus | $A_1$ | $A_2$ | F_$A_1$_ rice | F_$A_1$_ corn | Bonf | GOs | GO Description | Sequence Description |
| 175 | C | T | 0,386 | 0,097 | 3,21E-03 | GO:0004180,GO:0004185, GO:0006508,GO:0016020, GO:0016021,GO:0016021 | carboxypeptidase activity,serine-type carboxypeptidase activity,proteolysis,membrane,integral component of membrane,integral component of membrane | retinoid-inducible serine carboxypeptidase-like (680683) peptidase: hypothetical protein KGM_01563 (MER0350452:S10.UPW) PFAM: Serine carboxypeptidase(PF00450.18:Peptidase_S10) |
| 2571 | A | T | 1,000 | 0,358 | 6,42E-07 | GO:0004149,GO:0006099, GO:0006099,GO:0008152, GO:0016740,GO:0016746, GO:0045252,GO:0045252 | dihydrolipoyllysine-residue succinyltransferase activity,tricarboxylic acid cycle,tricarboxylic acid cycle,metabolic process,transferase activity,transferase activity, transferring acyl groups,oxoglutarate dehydrogenase complex,oxoglutarate dehydrogenase complex | dihydrolipoyllysine-residue succinyltransferase component of 2-oxoglutarate dehydrogenase complex, mitochondrial (7091) PFAM: 2-oxoacid dehydrogenases acyltransferase (catalytic domain)(PF00198.19:2-oxoacid_dh) |
| 2848 | C | T | 0,958 | 0,358 | 2,32E-08 | GO:0000166,GO:0003676, GO:0016779,GO:0006388, GO:0016740,GO:0016772, GO:0000166,GO:0003676, GO:0016779 | nucleotide binding,nucleic acid binding,nucleotidyltransferase activity,tRNA splicing, via endonucleolytic cleavage and ligation,transferase activity,transferase activity, transferring phosphorus-containing groups,nucleotide binding,nucleic acid | speckle targeted PIP5K1A-regulated poly(A) polymerase-like (680683) |

| | | | | | | | | |
|---|---|---|---|---|---|---|---|---|
| | | | | | | | binding,nucleotidyltransferase activity | |
| 4040 | C | T | 0,065 | 0,555 | 5,03E-03 | GO:0042302 | structural constituent of cuticle | calphotin-like (680683) |
| 4448 | G | A | 0,667 | 0,197 | 1,83E-05 | GO:0016491,GO:0016614, GO:0050660,GO:0055114, GO:0047875 | oxidoreductase activity,oxidoreductase activity, acting on CH-OH group of donors,flavin adenine dinucleotide binding,oxidation-reduction process,ecdysone oxidase activity | ecdysone oxidase (7091) PFAM: GMC oxidoreductase(PF00732.15:GMC_oxred_N) |
| 7156 | C | A | 0,932 | 0,025 | 4,55E-62 | GO:0003964,GO:0006278 | RNA-directed DNA polymerase activity,RNA-dependent DNA biosynthetic process | UNKNOWN ANNOTATION |
| 13970 | C | T | 0,500 | 0,080 | 3,98E-08 | GO:0035195,GO:0035068, GO:0035068 | gene silencing by miRNA,micro-ribonucleoprotein complex,micro-ribonucleoprotein complex | polyhomeotic-proximal chromatin protein-like (680683) |
| 15958 | C | T | 0,341 | 0,058 | 3,50E-03 | GO:0004180,GO:0004181, GO:0006508,GO:0008270 | carboxypeptidase activity,metallocarboxypeptidase activity,proteolysis,zinc ion binding | carboxypeptidase A2-like (7091) peptidase: hypothetical protein (MER0427121:M14.UNA) PFAM: Zinc carboxypeptidase(PF00246.20:Peptidase_M14 ) |
| 18557 | G | A | 0,341 | 0,054 | 2,05E-03 | GO:0005615,GO:0005615, GO:0006396,GO:0005730, GO:0005730 | extracellular space,extracellular space,RNA processing,nucleolus,nucleolus | serine protease inhibitor 9 (7091) inhibitor: antichymotrypsin precursor (MER0017977:I04.UPW) PFAM: Serpin (serine protease inhibitor)(PF00079.16:Serpin) |
| 19523 | T | C | 0,429 | 0,036 | 6,92E-07 | GO:0004672,GO:0004713, GO:0005524,GO:0006468, GO:0016020,GO:0016021, GO:0016021,GO:0016301, GO:0016310,GO:0018108 | protein kinase activity,protein tyrosine kinase activity,ATP binding,protein phosphorylation,membrane,integral component of membrane,integral component of membrane,kinase activity,phosphorylation,peptidyl-tyrosine phosphorylation | tyrosine-protein kinase transmembrane receptor Ror-like (680683) PFAM: Protein tyrosine kinase(PF07714.13:Pkinase_Tyr) |
| 21323 | A | G | 0,917 | 0,198 | 3,35E-06 | GO:0004252,GO:0006508, GO:0008233 | serine-type endopeptidase activity,proteolysis,peptidase activity | tryptase-like (680683) peptidase: serine proteinase-like protein (MER0090488:S01.936) |
| 24794 | T | A | 0,591 | 0,112 | 1,83E-09 | GO:0000166,GO:0003824, GO:0004356,GO:0005524, GO:0006542,GO:0006807, GO:0016874 | nucleotide binding,catalytic activity,glutamate-ammonia ligase activity,ATP binding,glutamine biosynthetic process,nitrogen compound metabolic process,ligase activity | glutamine synthetase 2 cytoplasmic (76193) PFAM: Glutamine synthetase, catalytic domain(PF00120.20:Gln-synt_C) |

| | | | | | | | | |
|---|---|---|---|---|---|---|---|---|
| 25212 | C | T | 0,500 | 0,121 | 3,91E-02 | GO:0003824,GO:0008483, GO:0009058,GO:0016740, GO:0030170 | catalytic activity,transaminase activity,biosynthetic process,transferase activity,pyridoxal phosphate binding | kynurenine/alpha-aminoadipate aminotransferase, mitochondrial (7091) |
| 28276 | A | G | 0,821 | 0,264 | 4,13E-04 | GO:0042302,GO:0016020, GO:0016021,GO:0016021, GO:0042302 | structural constituent of cuticle,membrane,integral component of membrane,integral component of membrane,structural constituent of cuticle | probable basic-leucine zipper transcription factor R (680683) |
| 29691 | T | A | 0,818 | 0,323 | 1,26E-03 | GO:0005215,GO:0005622, GO:0005622,GO:0006810 | transporter activity,intracellular,intracellular,transport | alpha-tocopherol transfer protein-like (680683) PFAM: CRAL/TRIO domain(PF00650.16:CRAL_TRIO) |
| 29909 | G | A | 0,900 | 0,005 | 1,50E-63 | GO:0000166,GO:0004672, GO:0004713,GO:0004714, GO:0005524,GO:0006468, GO:0007169,GO:0007169, GO:0016020,GO:0016021, GO:0016021,GO:0016301, GO:0016310,GO:0016740, GO:0018108 | nucleotide binding,protein kinase activity,protein tyrosine kinase activity,transmembrane receptor protein tyrosine kinase activity,ATP binding,protein phosphorylation,transmembrane receptor protein tyrosine kinase signaling pathway,transmembrane receptor protein tyrosine kinase signaling pathway,membrane,integral component of membrane,integral component of membrane,kinase activity,phosphorylation,transferase activity,peptidyl-tyrosine phosphorylation | epidermal growth factor receptor (76194) PFAM: Receptor L domain(PF01030.20:Recep_L_domain) |
| 29918 | G | T | 0,958 | 0,007 | 5,26E-79 | GO:0003824,GO:0004356, GO:0006542,GO:0006807, GO:0016874 | catalytic activity,glutamate-ammonia ligase activity,glutamine biosynthetic process,nitrogen compound metabolic process,ligase activity | glutamine synthetase 1, mitochondrial (680683) PFAM: Niemann-Pick C1 N terminus(PF16414.1:NPC1_N) |
| 36083 | T | C | 0,133 | 0,602 | 4,30E-02 | GO:0000166,GO:0005524 | nucleotide binding,ATP binding | uncharacterized LOC101738342 (7091) |
| 39360 | C | A | 0,591 | 0,018 | 6,84E-13 | GO:0016020,GO:0016021, GO:0016021 | membrane,integral component of membrane,integral component of membrane | uncharacterized LOC106142522 (680683) |
| 43057 | A | G | 0,067 | 0,578 | 4,48E-03 | GO:0005524,GO:0006457, GO:0006950,GO:0051082, GO:0005524,GO:0006457, GO:0006950,GO:0051082 | ATP binding,protein folding,response to stress,unfolded protein binding,ATP binding,protein folding,response to stress,unfolded protein binding | heat shock protein 83 (680683) PFAM: Hsp90 protein(PF00183.14:HSP90) |

| | | | | | | | | |
|---|---|---|---|---|---|---|---|---|
| **STRAIN** | | | | | | | | |
| Locus | $A_1$ | $A_2$ | F_$A_1$_ rice | F_$A_1$_ corn | Bonf | GOs | GO Description | Sequence Description |

| | | | | | | | | | | | |
|---|---|---|---|---|---|---|---|---|---|---|---|
| 8347 | A | T | 0,483 | 0,068 | 1,41E-16 | | | | GO:0007166,GO:0007166, GO:0030101,GO:0032393 | cell surface receptor signaling pathway,cell surface receptor signaling pathway,natural killer cell activation,MHC class I receptor activity | titin (680683) inhibitor: titin (MER0201921:I43.001) PFAM: Immunoglobulin I-set domain(PF07679.12:I-set) |
| 8935 | G | A | 0,317 | 0,114 | 4,77E-03 | | | | GO:0005509,GO:0005886, GO:0005886,GO:0007155, GO:0007156,GO:0016020, GO:0016021,GO:0016021 | calcium ion binding,plasma membrane,plasma membrane,cell adhesion,homophilic cell adhesion via plasma membrane adhesion molecules,membrane,integral component of membrane,integral component of membrane | cadherin-related tumor suppressor (66420) |
| 17464 | A | G | 0,550 | 0,024 | 2,69E-10 | | | | GO:0003964,GO:0004519, GO:0006278,GO:0090305, GO:0003676,GO:0008270 | RNA-directed DNA polymerase activity,endonuclease activity,RNA-dependent DNA biosynthetic process,nucleic acid phosphodiester bond hydrolysis,nucleic acid binding,zinc ion binding | gag-like protein (745177) |
| 23253 | C | G | 0,383 | 0,026 | 8,19E-14 | | | | GO:0000902,GO:0000902, GO:0005938,GO:0005938, GO:0030427,GO:0030427, GO:0031175,GO:0031175, GO:0090527,GO:0090527 | cell morphogenesis,cell morphogenesis,cell cortex,cell cortex,site of polarized growth,site of polarized growth,neuron projection development,neuron projection development,actin filament reorganization,actin filament reorganization | protein furry (7091) PFAM: Cell morphogenesis C-terminal(PF14225.2:MOR2-PAG1_C) |
| 56158 | C | A | 0,667 | 0,088 | 1,74E-02 | | | | GO:0035195,GO:0035068, GO:0035068 | gene silencing by miRNA,micro-ribonucleoprotein complex,micro-ribonucleoprotein complex | paramyosin (680683) |

| **HOST and STRAIN** | | | | | | | | | | | |
|---|---|---|---|---|---|---|---|---|---|---|---|
| | | | **Host** | | | **Strain** | | | | | |
| Locus | $A_1$ | $A_2$ | F_$A_1$_rice | F_$A_1$_corn | Bonf | F_$A_1$_rice | F_$A_1$_corn | Bonf | GOs | GO Description | Sequence Description |
| 1832 | A | G | 0,438 | 0,049 | 4,62E-02 | 0,317 | 0,011 | 2,76E-04 | GO:0003964,GO:0004519, GO:0006278,GO:0090305, GO:0003676,GO:0008270 | RNA-directed DNA polymerase activity,endonuclease activity,RNA-dependent DNA biosynthetic process,nucleic acid phosphodiester bond hydrolysis,nucleic acid binding,zinc ion binding | gag-like protein (745177) |
| 2384 | A | G | 0,354 | 0,016 | 4,05E-16 | 0,150 | 0,016 | 1,88E-04 | GO:0007165,GO:0007165 | signal transduction,signal transduction | sterile alpha and TIR motif-containing protein 1 (7091) |

| | | | | | | | | | | | |
|---|---|---|---|---|---|---|---|---|---|---|---|
| 3195 | A | T | 0,896 | 0,085 | 3,76E-33 | 0,432 | 0,078 | 2,02E-10 | GO:0031929,GO:0031929, GO:0031931,GO:0031931 | TOR signaling,TOR signaling,TORC1 complex,TORC1 complex | regulatory-associated protein of TOR (7091) |
| 3499 | A | G | 0,929 | 0,060 | 3,10E-39 | 0,407 | 0,057 | 6,73E-11 | GO:0003824,GO:0005975, GO:0016491,GO:0016616, GO:0019752,GO:0055114 | catalytic activity,carbohydrate metabolic process,oxidoreductase activity,oxidoreductase activity, acting on the CH-OH group of donors, NAD or NADP as acceptor,carboxylic acid metabolic process,oxidation-reduction process | malate dehydrogenase, mitochondrial-like (7091) PFAM: lactate/malate dehydrogenase, NAD binding domain(PF00056.19:Ldh_1_N) |
| 4867 | C | T | 0,875 | 0,005 | 1,44E-69 | 0,367 | 0,000 | 8,09E-20 | GO:0016020,GO:0016021, GO:0016021 | membrane,integral component of membrane,integral component of membrane | bestrophin-4 (7091) PFAM: Bestrophin, RFP-TM, chloride channel(PF01062.17:Bestrophin) |
| 5033 | G | C | 0,978 | 0,240 | 7,71E-14 | 0,622 | 0,256 | 1,56E-04 | GO:0000166,GO:0004672, GO:0004713,GO:0004714, GO:0005524,GO:0006468, GO:0007169,GO:0007169, GO:0016020,GO:0016021, GO:0016021,GO:0018108, GO:0016301,GO:0016310, GO:0016740,GO:0043548, GO:0043560,GO:0046777 | nucleotide binding,protein kinase activity,protein tyrosine kinase activity,transmembrane receptor protein tyrosine kinase activity,ATP binding,protein phosphorylation,transmembrane receptor protein tyrosine kinase signaling pathway,transmembrane receptor protein tyrosine kinase signaling pathway,membrane,integral component of membrane,integral component of membrane,peptidyl-tyrosine phosphorylation,kinase activity,phosphorylation,transferase activity,phosphatidylinositol 3-kinase binding,insulin receptor substrate binding,protein autophosphorylation | insulin receptor (7091) PFAM: Furin-like cysteine rich region(PF00757.16:Furin-like) |
| 9136 | G | A | 0,500 | 0,011 | 8,67E-12 | 0,196 | 0,014 | 1,66E-02 | GO:0035195,GO:0035068, GO:0035068,GO:0006401, GO:0006109,GO:0006109 | gene silencing by miRNA,micro-ribonucleoprotein complex,micro-ribonucleoprotein complex,RNA catabolic process,regulation of carbohydrate metabolic process,regulation of carbohydrate metabolic process | GG20431 gene product from transcript GG20431-RA (7220) |
| 10192 | G | A | 0,870 | 0,049 | 3,87E-40 | 0,395 | 0,043 | 1,67E-12 | GO:0016020,GO:0016021, GO:0016021,GO:0006355 | membrane,integral component of membrane,integral component of membrane,regulation of transcription, DNA-templated | uncharacterized LOC101737163 (7091) |

| | | | | | | | | | | | |
|---|---|---|---|---|---|---|---|---|---|---|---|
| 11124 | A | C | 0,938 | 0,015 | 5,28E-70 | 0,417 | 0,003 | 7,80E-24 | GO:0003824,GO:0004553, GO:0004559,GO:0005975, GO:0006013,GO:0008152, GO:0008270,GO:0015923, GO:0016020,GO:0016021, GO:0016021,GO:0016787, GO:0016798,GO:0030246, GO:0046872,GO:0000139, GO:0000139,GO:0006491, GO:0006517 | catalytic activity,hydrolase activity, hydrolyzing O-glycosyl compounds,alpha-mannosidase activity,carbohydrate metabolic process,mannose metabolic process,metabolic process,zinc ion binding,mannosidase activity,membrane,integral component of membrane,integral component of membrane,hydrolase activity,hydrolase activity, acting on glycosyl bonds,carbohydrate binding,metal ion binding,Golgi membrane,Golgi membrane,N-glycan processing,protein deglycosylation | alpha-mannosidase 2-like (7091) PFAM: Glycosyl hydrolases family 38 C-terminal domain(PF07748.9:Glyco_hydro_38C) |
| 12105 | G | A | 0,405 | 0,037 | 1,51E-10 | 0,209 | 0,025 | 4,54E-03 | GO:0003824,GO:0008483, GO:0009058,GO:0016740, GO:0030170 | catalytic activity,transaminase activity,biosynthetic process,transferase activity,pyridoxal phosphate binding | alanine aminotransferase 2 (66420) |
| 12314 | G | T | 0,979 | 0,232 | 6,44E-16 | 0,558 | 0,221 | 1,13E-07 | GO:0003824,GO:0009058, GO:0030170,GO:0008483, GO:0016740 | catalytic activity,biosynthetic process,pyridoxal phosphate binding,transaminase activity,transferase activity | alanine aminotransferase 1-like (76193) PFAM: Aminotransferase class I and II(PF00155.17:Aminotran_1_2) |
| 13075 | A | G | 0,452 | 0,000 | 8,69E-08 | 0,328 | 0,000 | 8,02E-06 | GO:0005509,GO:0005578, GO:0005578,GO:0007165, GO:0007165,GO:0001654, GO:0001654,GO:0005614, GO:0005614,GO:0050840, GO:0060173,GO:0060173 | calcium ion binding,proteinaceous extracellular matrix,proteinaceous extracellular matrix,signal transduction,signal transduction,eye development,eye development,interstitial matrix,interstitial matrix,extracellular matrix binding,limb development,limb development | SPARC-related modular calcium-binding protein 1 (76193) inhibitor: AGAP007489-PA (MER0023751:I31.UPW) PFAM: Thyroglobulin type-1 repeat(PF00086.14:Thyroglobulin_1) |
| 13433 | G | A | 0,958 | 0,023 | 3,86E-66 | 0,417 | 0,016 | 1,60E-19 | GO:0000166,GO:0005391, GO:0005524,GO:0006810, GO:0006811,GO:0006813, GO:0006814,GO:0010248, GO:0016020,GO:0016021, GO:0016021,GO:0016787, GO:0046872,GO:0090662 | nucleotide binding,sodium:potassium-exchanging ATPase activity,ATP binding,transport,ion transport,potassium ion transport,sodium ion transport,establishment or maintenance of transmembrane electrochemical gradient,membrane,integral component of membrane,integral component of membrane,hydrolase activity,metal ion binding,ATP hydrolysis coupled transmembrane transport | sodium/potassium-transporting ATPase subunit alpha-like (680683) PFAM: E1-E2 ATPase(PF00122.16:E1-E2_ATPase) |
| 13655 | G | A | 0,647 | 0,061 | 1,40E-15 | 0,384 | 0,027 | 1,18E-10 | GO:0003677,GO:0006313, GO:0015074 | DNA binding,transposition, DNA-mediated,DNA integration | uncharacterized LOC105557041 (411798) |

| | | | | | | | | | | | |
|---|---|---|---|---|---|---|---|---|---|---|---|
| 13911 | T | A | 0,796 | 0,018 | 5,14E-44 | 0,365 | 0,011 | 1,35E-15 | GO:0004757,GO:0006729, GO:0055114,GO:0016491 | sepiapterin reductase activity,tetrahydrobiopterin biosynthetic process,oxidation-reduction process,oxidoreductase activity | sepiapterin reductase (7091) PFAM: short chain dehydrogenase(PF00106.21:adh_short) |
| 15521 | T | C | 0,542 | 0,041 | 4,07E-19 | 0,266 | 0,028 | 1,42E-07 | GO:0005622,GO:0005622, GO:0006886,GO:0008536 | intracellular,intracellular,intracellular protein transport,Ran GTPase binding | exportin-1 (66420) PFAM: CRM1 C terminal(PF08767.7:CRM1_C) |
| 20774 | G | A | 0,688 | 0,131 | 2,69E-08 | 0,354 | 0,119 | 1,41E-03 | GO:0005615,GO:0005615, GO:0006508,GO:0008233, GO:0016020,GO:0016021, GO:0016021 | extracellular space,extracellular space,proteolysis,peptidase activity,membrane,integral component of membrane,integral component of membrane | uncharacterized LOC106131445 (680683) inhibitor: serpin peptidase inhibitor 5 (MER0181019:I04.UPW) PFAM: Serpin (serine protease inhibitor)(PF00079.16:Serpin) |
| 21761 | T | G | 0,478 | 0,003 | 9,70E-33 | 0,192 | 0,000 | 8,07E-09 | GO:0000015,GO:0000015, GO:0000287,GO:0004634, GO:0006096,GO:0046983, GO:0016829 | phosphopyruvate hydratase complex,phosphopyruvate hydratase complex,magnesium ion binding,phosphopyruvate hydratase activity,glycolytic process,protein dimerization activity,lyase activity | enolase-like (680683) PFAM: Enolase, C-terminal TIM barrel domain(PF00113.18:Enolase_C) |
| 22209 | C | T | 0,714 | 0,008 | 2,70E-47 | 0,286 | 0,003 | 5,86E-14 | GO:0006511,GO:0006511, GO:0016579,GO:0036459, GO:0016573,GO:0016573, GO:0031935,GO:0031935, GO:0032153,GO:0032153, GO:0034613,GO:0035616, GO:0035616,GO:0045859, GO:0045859,GO:0048316, GO:0048316,GO:0051286, GO:0051286,GO:0060628 | ubiquitin-dependent protein catabolic process,ubiquitin-dependent protein catabolic process,protein deubiquitination,thiol-dependent ubiquitinyl hydrolase activity,histone acetylation,histone acetylation,regulation of chromatin silencing,regulation of chromatin silencing,cell division site,cell division site,cellular protein localization,histone H2B conserved C-terminal lysine deubiquitination,histone H2B conserved C-terminal lysine deubiquitination,regulation of protein kinase activity,regulation of protein kinase activity,seed development,seed development,cell tip,cell tip,regulation of ER to Golgi vesicle-mediated transport | ubiquitin carboxyl-terminal hydrolase 35 (680683) peptidase: hypothetical protein (MER0608825:C19.UPW) PFAM: Ubiquitin carboxyl-terminal hydrolase(PF00443.25:UCH) |
| 22419 | G | A | 0,929 | 0,044 | 1,12E-24 | 0,438 | 0,042 | 3,92E-10 | GO:0003824,GO:0003867, GO:0008483,GO:0009448, GO:0016740,GO:0030170, GO:0035195,GO:0035068, GO:0035068 | catalytic activity,4-aminobutyrate transaminase activity,transaminase activity,gamma-aminobutyric acid metabolic process,transferase activity,pyridoxal phosphate binding,gene silencing by miRNA,micro-ribonucleoprotein complex,micro-ribonucleoprotein complex | 4-aminobutyrate aminotransferase, mitochondrial (76193) PFAM: Aminotransferase class-III(PF00202.17:Aminotran_3) |
| 23974 | C | G | 0,682 | 0,005 | 2,54E-48 | 0,267 | 0,003 | 3,49E-13 | GO:0046872 | metal ion binding | uncharacterized LOC101743416 (7091) |

| | | | | | | | | | | |
|---|---|---|---|---|---|---|---|---|---|---|
| 25368 | T | C | 0,917 | 0,043 | 4,15E-52 | 0,414 | 0,040 | 1,70E-15 | GO:0000166,GO:0004672, GO:0004713,GO:0005003, GO:0005524,GO:0005887, GO:0005887,GO:0006468, GO:0007169,GO:0007169, GO:0016020,GO:0016021, GO:0016021,GO:0016301, GO:0016310,GO:0016740, GO:0018108,GO:0048013, GO:0048013 | nucleotide binding,protein kinase activity,protein tyrosine kinase activity,ephrin receptor activity,ATP binding,integral component of plasma membrane,integral component of plasma membrane,protein phosphorylation,transmembrane receptor protein tyrosine kinase signaling pathway,transmembrane receptor protein tyrosine kinase signaling pathway,membrane,integral component of membrane,integral component of membrane,kinase activity,phosphorylation,transferase activity,peptidyl-tyrosine phosphorylation,ephrin receptor signaling pathway,ephrin receptor signaling pathway | ephrin type-B receptor 1-B (51655) PFAM: Ephrin receptor ligand binding domain(PF01404.15:Ephrin_lbd) |
| 25469 | A | G | 0,767 | 0,037 | 6,12E-30 | 0,281 | 0,031 | 2,09E-06 | GO:0006887,GO:0016020 | exocytosis,membrane | synaptotagmin-7 (680683) PFAM: C2 domain(PF00168.26:C2) |
| 26996 | C | G | 0,425 | 0,046 | 9,12E-10 | 0,202 | 0,042 | 2,69E-03 | GO:0000166,GO:0002161, GO:0004812,GO:0004832, GO:0005524,GO:0006412, GO:0006418,GO:0006438, GO:0006450,GO:0006450, GO:0016874,GO:0000166, GO:0002161,GO:0004812, GO:0004832,GO:0005524, GO:0006418,GO:0006438, GO:0006450,GO:0006450, GO:0006412,GO:0016874 | nucleotide binding,aminoacyl-tRNA editing activity,aminoacyl-tRNA ligase activity,valine-tRNA ligase activity,ATP binding,translation,tRNA aminoacylation for protein translation,valyl-tRNA aminoacylation,regulation of translational fidelity,regulation of translational fidelity,ligase activity,nucleotide binding,aminoacyl-tRNA editing activity,aminoacyl-tRNA ligase activity,valine-tRNA ligase activity,ATP binding,tRNA aminoacylation for protein translation,valyl-tRNA aminoacylation,regulation of translational fidelity,regulation of translational fidelity,translation,ligase activity | valine--tRNA ligase (7091) PFAM: Anticodon-binding domain of tRNA(PF08264.9:Anticodon_1) |
| 27191 | T | C | 0,605 | 0,024 | 1,17E-21 | 0,318 | 0,008 | 4,91E-11 | GO:0003676,GO:0008270, GO:0015074 | nucleic acid binding,zinc ion binding,DNA integration | UNKNOWN ANNOTATION |
| 27272 | C | T | 0,935 | 0,008 | 7,71E-73 | 0,388 | 0,003 | 1,13E-20 | GO:0007165,GO:0007165 | signal transduction,signal transduction | rho GTPase-activating protein 1-like (680683) PFAM: Divergent CRAL/TRIO domain(PF13716.2:CRAL_TRIO_2) |
| 27490 | C | G | 0,900 | 0,118 | 1,16E-17 | 0,553 | 0,109 | 3,66E-08 | GO:0005509,GO:0006897 | calcium ion binding,endocytosis | epidermal growth factor receptor substrate 15-like 1 (66420) PFAM: Cytoskeletal-regulatory complex EF hand(PF12763.3:EF-hand_4) |

| | | | | | | | | | | | |
|---|---|---|---|---|---|---|---|---|---|---|---|
| 27957 | G | T | 0,844 | 0,073 | 4,40E-18 | 0,513 | 0,027 | 5,85E-15 | GO:0003676,GO:0008270, GO:0015074 | nucleic acid binding,zinc ion binding,DNA integration | uncharacterized protein K02A2.6-like (411798) |
| 28345 | C | T | 0,891 | 0,038 | 1,49E-48 | 0,397 | 0,030 | 3,26E-16 | GO:0005509,GO:0016020, GO:0016021,GO:0016021 | calcium ion binding,membrane,integral component of membrane,integral component of membrane | low-density lipoprotein receptor-related protein 2 (51655) |
| 28980 | A | G | 0,583 | 0,120 | 1,14E-02 | 0,347 | 0,099 | 2,13E-03 | GO:0003676,GO:0008270, GO:0015074,GO:0046872 | nucleic acid binding,zinc ion binding,DNA integration,metal ion binding | uncharacterized LOC105388554 (51655) PFAM: Integrase core domain(PF00665.22:rve) |
| 29602 | G | C | 0,692 | 0,007 | 3,88E-33 | 0,243 | 0,008 | 2,86E-06 | GO:0016740,GO:0004364 | transferase activity,glutathione transferase activity | glutathione S-transferase sigma 1 (7091) |
| 30789 | A | T | 0,967 | 0,104 | 4,57E-24 | 0,375 | 0,104 | 4,36E-03 | GO:0005198,GO:0005737, GO:0005737,GO:0006810, GO:0006886,GO:0015031, GO:0016020,GO:0016192, GO:0030117,GO:0030126, GO:0030126,GO:0000139, GO:0000139,GO:0005794, GO:0005794,GO:0030663, GO:0030663,GO:0031410, GO:0019028,GO:0019028, GO:0000139,GO:0000139, GO:0005198,GO:0005737, GO:0005737,GO:0005794, GO:0005794,GO:0006810, GO:0006886,GO:0015031, GO:0016020,GO:0016192, GO:0030117,GO:0030126, GO:0030126,GO:0030663, GO:0030663,GO:0031410, GO:0019028,GO:0019028 | structural molecule activity,cytoplasm,cytoplasm,transport,intracellular protein transport,protein transport,membrane,vesicle-mediated transport,membrane coat,COPI vesicle coat,COPI vesicle coat,Golgi membrane,Golgi membrane,Golgi apparatus,Golgi apparatus,COPI-coated vesicle membrane,COPI-coated vesicle membrane,cytoplasmic vesicle,viral capsid,viral capsid,Golgi membrane,Golgi membrane,structural molecule activity,cytoplasm,cytoplasm,Golgi apparatus,Golgi apparatus,transport,intracellular protein transport,protein transport,membrane,vesicle-mediated transport,membrane coat,COPI vesicle coat,COPI vesicle coat,COPI-coated vesicle membrane,COPI-coated vesicle membrane,cytoplasmic vesicle,viral capsid,viral capsid | nonclathrin coat protein gamma1-COP (7091) PFAM: Adaptin N terminal region(PF01602.16:Adaptin_N) |
| 31363 | G | A | 0,889 | 0,095 | 8,60E-24 | 0,427 | 0,083 | 5,63E-08 | GO:0004553,GO:0005975, GO:0008152,GO:0016787, GO:0016798,GO:0004553, GO:0005975,GO:0008152, GO:0016787,GO:0016798 | hydrolase activity, hydrolyzing O-glycosyl compounds,carbohydrate metabolic process,metabolic process,hydrolase activity,hydrolase activity, acting on glycosyl bonds,hydrolase activity, hydrolyzing O-glycosyl compounds,carbohydrate metabolic process,metabolic process,hydrolase activity,hydrolase activity, acting on glycosyl bonds | myrosinase 1-like (76194) PFAM: Glycosyl hydrolase family 1(PF00232.14:Glyco_hydro_1) |

| | | | | | | | | | | | |
|---|---|---|---|---|---|---|---|---|---|---|---|
| 36236 | G | A | 0,400 | 0,020 | 3,79E-13 | 0,149 | 0,017 | 3,00E-03 | GO:0003824,GO:0008152, GO:0016787 | catalytic activity,metabolic process,hydrolase activity | fumarylacetoacetate hydrolase domain-containing protein 2 (66420) PFAM: Fumarylacetoacetate (FAA) hydrolase family(PF01557.14:FAA_hydrolase) |
| 36473 | G | A | 0,719 | 0,037 | 2,24E-23 | 0,355 | 0,028 | 1,70E-09 | GO:0003676,GO:0004523, GO:0090502 | nucleic acid binding,RNA-DNA hybrid ribonuclease activity,RNA phosphodiester bond hydrolysis, endonucleolytic | uncharacterized LOC106709759 (76193) PFAM: Endonuclease-reverse transcriptase (PF14529.2:Exo_endo_phos_2) |
| 40681 | A | C | 0,821 | 0,303 | 1,76E-02 | 0,833 | 0,284 | 2,44E-03 | GO:0005576,GO:0005615, GO:0005615,GO:0004866, GO:0010951 | extracellular region,extracellular space,extracellular space,endopeptidase inhibitor activity,negative regulation of endopeptidase activity | alpha-2-macroglobulin-like (7091) inhibitor: alpha 2-macroglobulin (MER0177021:I39.UPW) PFAM: A-macroglobulin complement component(PF07678.10:A2M_comp) |
| 47790 | A | G | 0,625 | 0,036 | 3,62E-14 | 0,243 | 0,029 | 9,29E-04 | GO:0006417,GO:0006417, GO:0019887,GO:0019901, GO:0033554,GO:0033674, GO:0033674,GO:0043022, GO:0045859,GO:0045859, GO:0005634,GO:0005634 | regulation of translation,regulation of translation,protein kinase regulator activity,protein kinase binding,cellular response to stress,positive regulation of kinase activity,positive regulation of kinase activity,ribosome binding,regulation of protein kinase activity,regulation of protein kinase activity,nucleus,nucleus | translational activator GCN1 (7091) PFAM: Domain of unknown function (DUF3554)(PF12074.4:DUF3554) |
