# Supplementary material for: Loci under selection and markers associated with host plant and host-related strains shape the genetic structure of Brazilian populations of Spodoptera frugiperda (Lepidoptera, Noctuidae)
Source: PLoS One. 2018 May 22;13(5):e0197378. doi: 10.1371/journal.pone.0197378 (PMC5963752; doi:10.1371/journal.pone.0197378)
Supplement: S3 Table — Gene Ontology (GO) annotation and description of loci that contributed to the arrangement of individuals in three clusters found in the DAPC analysis. † Alleles fixed in cluster 3, composed of individuals from R-RS. ‡ Aspect: M = Molecular function, B = Biological process, C = Cellular component. (PDF) [file pone.0197378.s003.pdf]

Markers associated with host plant and host-related strains and the genetic structure of Brazilian populations of *Spodoptera frugiperda* (Lepidoptera, Noctuidae)

Karina Lucas Silva-Brandão, Aline Peruchi, Noemy Seraphim, Natália Faraj Murad, Renato Assis Carvalho, Juliano Ricardo Farias, Celso Omoto, Fernando Luis Cônsoli, Antonio Figueira, Marcelo Mendes Brandão

Supporting Information

S3 Table. Gene Ontology (GO) annotation and description of loci that contributed to the arrangement of individuals in three clusters found in the DAPC analysis. † Alleles fixed in cluster 3, composed of individuals from R-RS. ‡ Aspect: M = Molecular function, B = Biological process, C = Cellular component.

Locus	Description	GO Description, aspect‡ and ID	SpodoBase Annotation
2848	speckle targeted PIP5K1A-regulated poly(A) polymerase-like (680683)	nucleotide binding (M, GO:0000166); nucleic acid binding (M, GO:0003676); nucleotidyltransferase activity (M, GO:0016779); transferase activity (M, GO:0016740); tRNA splicing (B, GO:0006388)	putative terminal uridylyl transferase 1, U6 snRNA-specific-like protein
3499†	malate dehydrogenase, mitochondrial-like (7091) PFAM: lactate/malate dehydrogenase, NAD binding domain (PF00056.19:Ldh_1_N)	carbohydrate metabolic process (B, GO:0005975); carboxylic acid metabolic process (B, GO:0019752); oxidation-reduction process (B, GO:0055114); catalytic activity (M, GO:0003824); oxidoreductase activity (M, GO:0016491)	putative malate dehydrogenase
4867†	bestrophin-4 (7091) PFAM: Bestrophin, RFP-TM, chloride channel (PF01062.17:Bestrophin)	integral component of membrane (C, GO:0016021)	-
5033	insulin receptor (7091) PFAM: Furin-like cysteine rich region (PF00757.16:Furin-like)	nucleotide binding (M, GO:0000166); protein kinase activity (M, GO:0004672); ATP binding (M, GO:0005524); protein phosphorylation (B, GO:0006468)	-
7156†	UNKNOWN ANNOTATION	RNA-directed DNA polymerase activity (M, GO:0003964); RNA-dependent DNA biosynthetic process (B, GO:0006278)	-
7894†	sepiapterin reductase (7091) PFAM: short chain dehydrogenase (PF00106.21:adh_short)	tetrahydrobiopterin biosynthetic process (B, GO:0006729); oxidation-reduction process (B, GO:0055114); oxidoreductase activity (M, GO:0016491)	-
11124†	alpha-mannosidase 2-like (7091) PFAM: Glycosyl hydrolases family 38 C-terminal domain (PF07748.9:Glyco_hydro_38C)	carbohydrate metabolic process (B, GO:0005975); mannose metabolic process (B, GO:0006013); metabolic process (B, GO:0008152); integral component of membrane (C, GO:0016021); Golgi membrane (C, GO:0000139); N-glycan processing (B, GO:0006491); protein deglycosylation (B, GO:0006517); catalytic activity (M, GO:0003824); hydrolase activity (M, GO:0004553); metal ion binding (M, GO:0046872)	-
13433†	sodium/potassium-transporting ATPase subunit alpha-like (680683) PFAM: E1-E2 ATPase (PF00122.16:E1-E2_ATPase)	nucleotide binding (M, GO:0000166); sodium:potassium-exchanging ATPase activity (M, GO:0005391); ATP binding (M, GO:0005524); hydrolase activity (M, GO:0016787); metal ion binding (M, GO:0046872); ion transport (B, GO:0006811); establishment or maintenance of transmembrane electrochemical gradient (B, GO:0010248)	-
14975	cadherin-related tumor suppressor (76193) peptidase: hypothetical protein (MER0256887:U69.UPW) PFAM: Cadherin domain (PF00028.13:Cadherin)	calcium ion binding (M, GO:0005509); plasma membrane (C, GO:0005886); cell adhesion (B, GO:0007155); integral component of membrane (C, GO:0016021)	-
23253†	protein furry (7091) PFAM: Cell morphogenesis C-terminal (PF14225.2:MOR2-PAG1_C)	cell morphogenesis (B, GO:0000902); cell cortex (C, GO:0005938); site of polarized growth (C, GO:0030427); neuron projection development (B, GO:0031175); actin filament reorganization (B, GO:0090527)	-
25368†	ephrin type-B receptor 1-B (51655) PFAM: Ephrin receptor ligand binding domain (PF01404.15:Ephrin_lbd)	nucleotide binding (M, GO:0000166); protein kinase activity (M, GO:0004672); protein tyrosine kinase activity (M, GO:0004713); ephrin receptor activity (M, GO:0005003); ATP binding (M, GO:0005524); transferase activity (M, GO:0016740); integral component of plasma membrane (C, GO:0005887); protein phosphorylation (B, GO:0006468); integral component of membrane (C, GO:0016021); phosphorylation (B, GO:0016310); ephrin receptor signaling	-

		pathway (B, GO:0048013)	
25575	serine/threonine-protein kinase SIK2-like (680683) PFAM: Protein kinase domain (PF00069.21:Pkinase)	phosphorylation (B, GO:0016310); kinase activity (M, GO:0016301); ATP binding (M, GO:0005524); nucleotide binding (M, GO:0000166)	-
27272†	rho GTPase-activating protein 1-like (680683) PFAM: Divergent CRAL/TRIO domain (PF13716.2:CRAL_TRIO_2)	signal transduction (B, GO:0007165)	-
29918†	glutamine synthetase 1, mitochondrial (680683) PFAM: Niemann-Pick C1 N terminus (PF16414.1:NPC1_N)	glutamine biosynthetic process (B, GO:0006542); nitrogen compound metabolic process (B, GO:0006807); catalytic activity (M, GO:0003824); glutamate-ammonia ligase activity (M, GO:0004356); ligase activity (M, GO:0016874)	-
