# Supplementary material for: Loci under selection and markers associated with host plant and host-related strains shape the genetic structure of Brazilian populations of Spodoptera frugiperda (Lepidoptera, Noctuidae)
Source: PLoS One. 2018 May 22;13(5):e0197378. doi: 10.1371/journal.pone.0197378 (PMC5963752; doi:10.1371/journal.pone.0197378)
Supplement: S1 Fig — Sequence Spodoptera litura ABCC1-like (KM453742) was the best match when BLAST was used (Identities 1477/1635, 90%); sequence Spodoptera exigua ABCC2 (KM068116) is discussed in [126]; sequence Spodoptera exigua ABCC3 (KF926101) discussed in [83]; sequence Chrysomela tremula ABCB1 (KX686490) is discussed in [86]; sequence Helicoverpa armigera ABCA2 (KP259911) is annotated as in [85]. TpM = transporter motif (in blue); ATP = ATP-binding domains (in red); region of overlap with locus 12150 (in green), with the amino acid related to the polymorphism in the position 896 of the alignment (amino acid Leucine in the contig 12636, and polymorphism C/TTG in the locus 12150). Figure generated in Geneious v. 10.2 (Biomatters). (PDF) [file pone.0197378.s004.pdf]

Markers associated with host plant and host-related strains and the genetic structure of Brazilian populations of *Spodoptera frugiperda* (Lepidoptera, Noctuidae)

Karina Lucas Silva-Brandão, Aline Peruchi, Noemy Seraphim, Natália Faraj Murad, Renato Assis Carvalho, Juliano Ricardo Farias, Celso Omoto, Fernando Luis Cônsoli, Antonio Figueira, Marcelo Mendes Brandão

Supporting Information

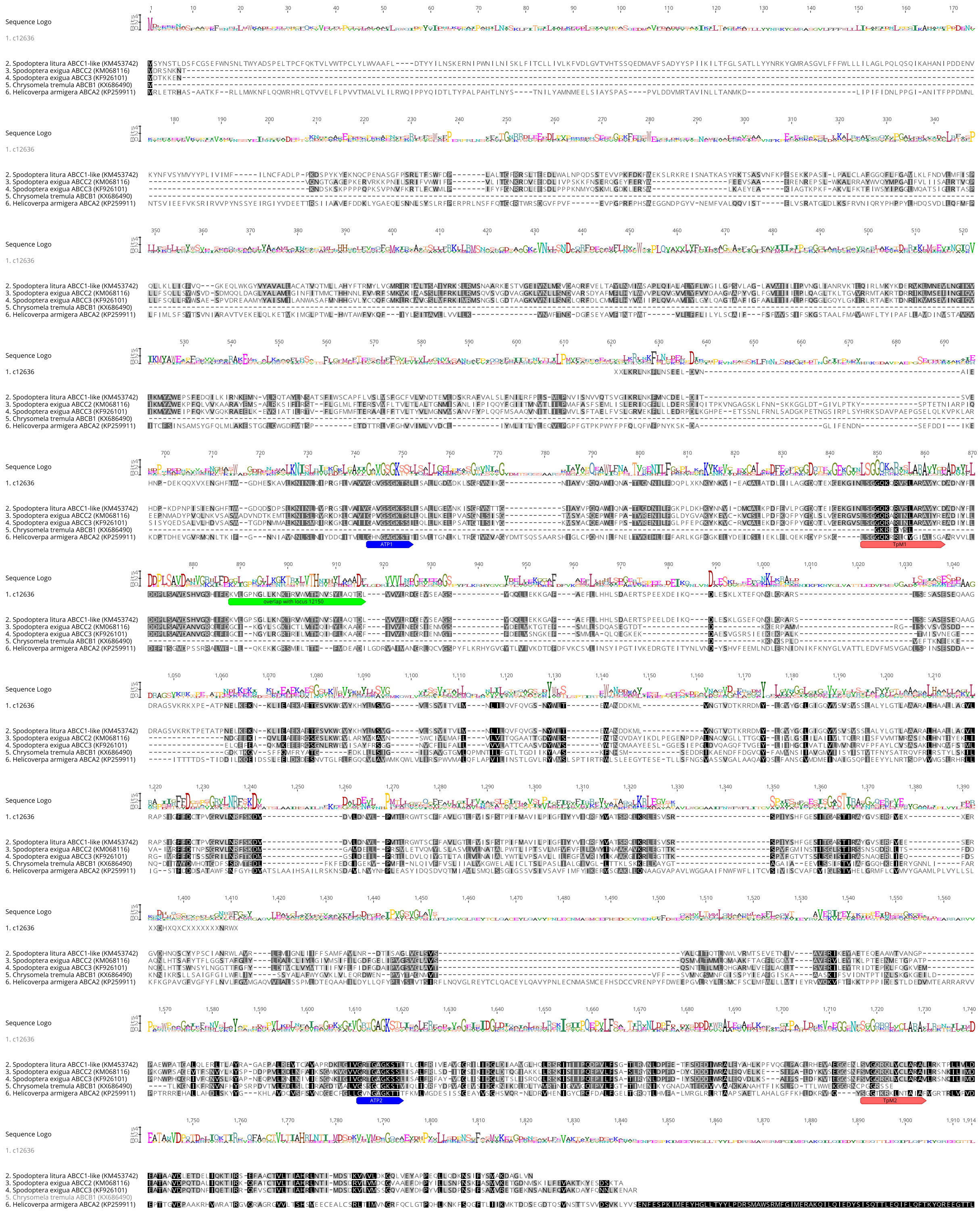
Sequence Logo
1. c12636
2. Spodoptera litura ABCC1-like (KM453742)
3. Spodoptera exigua ABCC2 (KM068116)
4. Spodoptera exigua ABCC3 (KF926101)
5. Chrysomela tremula ABCB1 (KX686490)
6. Helicoverpa armigera ABCA2 (KP259911)
ATP1
TpM1
overlap with locus 12150
ATP2
TpM2

S1 Fig. Alignment of contig 12636 with sequences of ABC proteins from the literature. Sequence *Spodoptera litura* ABCC1-like (KM453742) was the best match when BLAST was used (Identities 1477/1635, 90%); sequence *Spodoptera exigua* ABCC2 (KM068116) is discussed in [1]; sequence *Spodoptera exigua* ABCC3 (KF926101) discussed in [2]; sequence Chrysomela tremula ABCB1 (KX686490) is discussed in [3]; sequence *Helicoverpa armigera* ABCA2 (KP259911) is annotated as in [4]. TpM = transporter motif (in blue); ATP = ATP-binding domains (in red); region of overlap with locus 12150 (in green), with the amino acid related to the polymorphism in the position 896 of the alignment (amino acid Leucine in the contig 12636, and polymorphism C/TTG in the locus 12150). Figure generated in Geneious v. 10.2 (Biomatters).

References

1. Ren XL, Jiang WL, Ma YJ, Hu HY, Ma XY, Ma Y, et al. The *Spodoptera exigua* (Lepidoptera: Noctuidae) ABCC2 Mediates Cry1Ac Cytotoxicity and, in Conjunction with Cadherin, Contributes to Enhance Cry1Ca Toxicity in Sf9 Cells. J Econ Entomol. 2016;109(6):2281-9.

2. Park Y, Gonzalez-Martinez RM, Navarro-Cerrillo G, Chakroun M, Kim Y, Ziarsolo P, et al. ABCC transporters mediate insect resistance to multiple Bt toxins revealed by bulk segregant analysis. BMC biology. 2014;12:46.

3. Pauchet Y, Bretschneider A, Augustin S, Heckel DG. A P-Glycoprotein is linked to resistance to the *Bacillus thuringiensis* Cry3Aa toxin in a leaf beetle. Toxins. 2016;8(12).

4. Tay WT, Mahon RJ, Heckel DG, Walsh TK, Downes S, James WJ, et al. Insect resistance to *Bacillus thuringiensis* toxin Cry2Ab Is conferred by mutations in an ABC transporter subfamily A protein. Plos Genet. 2015;11(11):e1005534.
