# Supplementary material for: Loci under selection and markers associated with host plant and host-related strains shape the genetic structure of Brazilian populations of Spodoptera frugiperda (Lepidoptera, Noctuidae)
Source: PLoS One. 2018 May 22;13(5):e0197378. doi: 10.1371/journal.pone.0197378 (PMC5963752; doi:10.1371/journal.pone.0197378)
Supplement: S5 Fig — Sequence Helicoverpa armigera (LOC 110371604) is a predicted cadherin-related tumor suppressor; sequence Helicoverpa armigera (BtR) (AY647974) is annotated as in [121], and additional information [127, 128]; sequence Manduca sexta (BT-R1) (AF319973) is annotated as in [122]. EC = cadherin repeats (in red); TBR = putative Cry1Ac toxin binding region (in blue). Figure generated in Geneious v. 10.2 (Biomatters). (PDF) [file pone.0197378.s008.pdf]

Markers associated with host plant and host-related strains and the genetic structure of Brazilian populations of *Spodoptera frugiperda* (Lepidoptera, Noctuidae)

Karina Lucas Silva-Brandão, Aline Peruchi, Noemy Seraphim, Natália Faraj Murad, Renato Assis Carvalho, Juliano Ricardo Farias, Celso Omoto, Fernando Luis Cônsoli, Antonio Figueira, Marcelo Mendes Brandão

Supporting Information

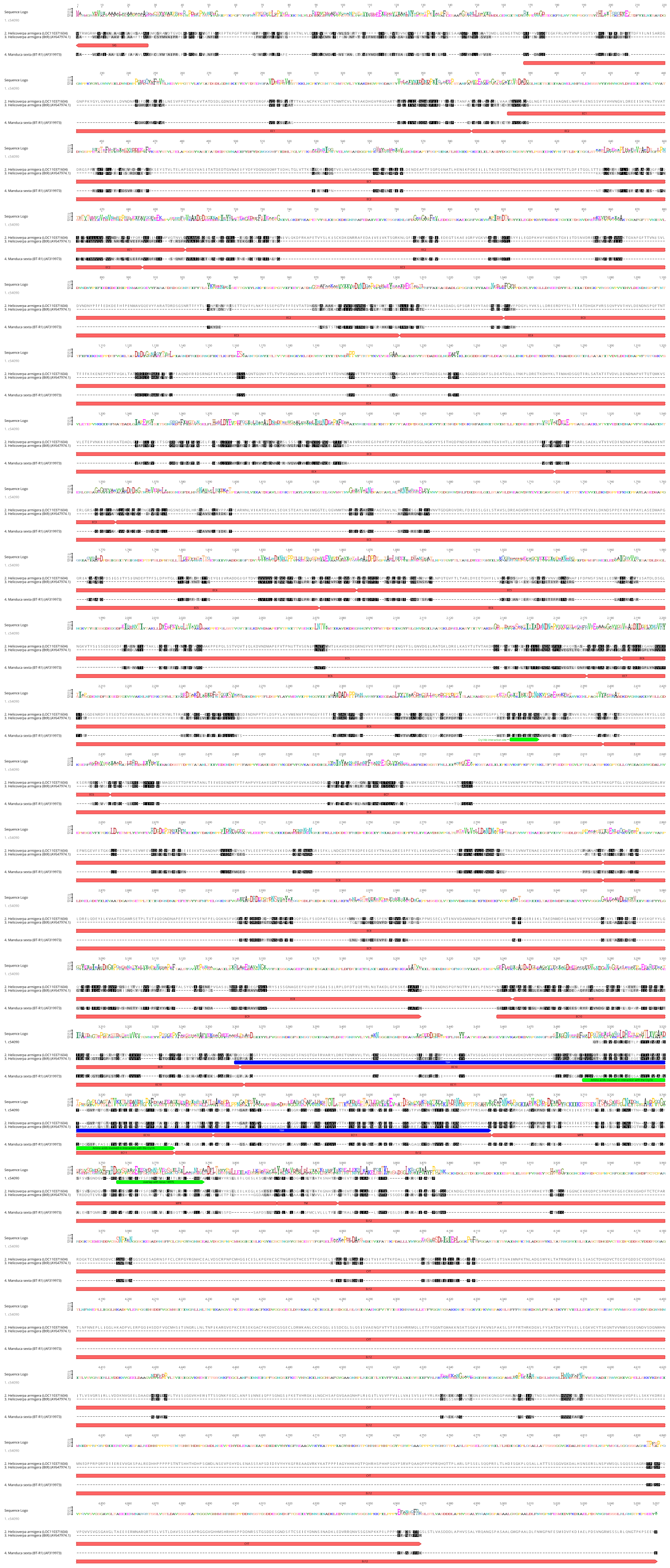
Sequence Logo
1. c54090
2. Helicoverpa armigera (LOC110371604)
3. Helicoverpa armigera (BtR) (AY647974.1)
4. Manduca sexta (BT-R1) (AF319973)
SIG
EC1

S5 Fig. Alignment of contig 54090 with sequences of cadherin proteins from the literature. Sequence *Helicoverpa armigera* (LOC 110371604) is a predicted cadherin-related tumor suppressor; sequence *Helicoverpa armigera* (BtR) (AY647974) is annotated as in [1], and additional information [2, 3]; sequence *Manduca sexta* (BT-R1) (AF319973) is annotated as in [4]. EC = cadherin repeats (in red); TBR = putative Cry1Ac toxin binding region (in blue). Figure generated in Geneious v. 10.2 (Biomatters).

References

1. Xu XJ, Yu LY, Wu YD. Disruption of a cadherin gene associated with resistance to Cry1Ac delta-endotoxin of *Bacillus thuringiensis* in *Helicoverpa armigera*. Applied and Environmental Microbiology. 2005;71(2):948-54.
2. Wang G, Wu K, Liang G, Guo Y. Gene cloning and expression of cadherin in midgut of *Helicoverpa armigera* and its Cry1A binding region. Science in China Series C, Life sciences. 2005;48(4):346-56.
3. Zhang H, Yu S, Shi Y, Yang Y, Fabrick JA, Wu Y. Intra- and extracellular domains of the *Helicoverpa armigera* cadherin mediate Cry1Ac cytotoxicity. Insect Biochem Mol Biol. 2017;86:41-9.
4. Dorsch JA, Candas M, Griko NB, Maaty WS, Midboe EG, Vadlamudi RK, et al. Cry1A toxins of *Bacillus thuringiensis* bind specifically to a region adjacent to the membrane-proximal extracellular domain of BT-R(1) in *Manduca sexta*: involvement of a cadherin in the entomopathogenicity of *Bacillus thuringiensis*. Insect Biochem Mol Biol. 2002;32(9):1025-36.
